# Rathke’s cleft cyst: From history to molecular genetics

**DOI:** 10.1007/s11154-025-09949-6

**Published:** 2025-02-13

**Authors:** Aysa Hacioglu, Halil Tekiner, Meric A. Altinoz, Gazanfer Ekinci, Jean-François Bonneville, Kaan Yaltirik, Aydin Sav, Ugur Ture, Fahrettin Kelestimur

**Affiliations:** 1 Department of Endocrinology, Erciyes University, Kayseri, Turkey; 2 Department of Medical History, Erciyes University, Kayseri, Turkey; 3 Department of Biochemistry, Acibadem University, Istanbul, Turkey; 4 Department of Radiology, Yeditepe University, Istanbul, Turkey; 5 Departments of Medical Imaging and Endocrinology, Centre Hospitalier Universitaire de Liège, Liège, Belgium; 6 Department of Neurosurgery, Yeditepe University, Istanbul, Turkey; 7 Department of Pathology, Yeditepe University, Istanbul, Turkey; 8 Department of Endocrinology, Yeditepe University School of Medicine, Istanbul, Turkey

**Keywords:** Rathke, Luschka, Cleft, Cyst, Pituitary, Hypopituitarism

## Abstract

A Rathke’s cleft cyst (RCC) is a remnant of the embryologic Rathke’s pouch and a common pituitary lesion. A true RCC is lined with ciliated cuboidal or columnar epithelia with occasional goblet cells and squamous metaplasia. A RCC is frequently diagnosed incidentally through magnetic resonance imaging and computed tomography of the brain or pituitary gland. Presentation can range from an asymptomatic clinical picture to a rapidly progressive disease. RCC are located most often in the sellar and suprasellar regions and a careful differential diagnosis is crucial, especially to exclude craniophryngioma. Recent studies illuminate novel molecular mechanisms and markers for understanding the pathogenesis of RCC. PROP-1, a paired-like homeodomain transcription factor, controls pituitary ontogeny and its high expression induces RCCs. Both transgenic mouse models and immunohistochemical analysis of human RCCs indicate that the leukemia inhibitory factor is involved in pathogenesis. The expression of cytokeratins 8 and 2 in RCCs, but not in craniopharyngiomas, and the presence of beta-catenin mutations in many craniopharyngiomas, but not in RCCs, help with the differential diagnosis. For asymptomatic and small RCCs, observation is appropriate, with serial magnetic resonance imaging and hormonal investigation depending on the patient’s clinical status. Surgical resection may be required for symptomatic RCC and recurrence rates are generally low. For patients with a recurrence, stereotactic radiosurgery is an effective approach with low risk.

## Introduction

Martin Heinrich Rathke (1793–1860), a pioneering German anatomist and embryologist, first described Rathke’s pouch in 1838 [1]. Also known as Rathke’s pocket or Rathke’s diverticulum, this ectodermal structure arises from the upper surface of the embryonic stomodaeum and plays a crucial role in pituitary gland development [2, 3]. During fetal development, a remnant known as Rathke’s cleft forms due to cellular proliferation in the pouch’s anterior wall [2]. Rathke’s cleft cyst (RCC) is a benign, epithelium-lined cyst typically located in the sellar and/or suprasellar regions. It originates from these remnants and contains cerebrospinal fluid or mucoid material rich in cholesterol and proteins [4].

While RCCs occasionally occur in children, they are more common in adults and rank among the most frequent pituitary lesions encountered in endocrinology and neurosurgery. Most RCCs are incidentally discovered during imaging studies, such as magnetic resonance imaging (MRI) and computed tomography (CT) scans, performed for unrelated reasons. Symptoms typically arise when cyst enlargement causes local mass effects on adjacent structures, such as the pituitary gland or optic apparatus. Symptomatic manifestations include headaches, vision abnormalities, hypopituitarism, galactorrhea due to hyperprolactinemia, central diabetes insipidus, or, rarely, cyst infection [5].

Recent molecular studies have enhanced the understanding of RCC pathogenesis, suggesting that future management, particularly in recurrent cases, may integrate surgical and targeted molecular therapies. In a retrospective study of 105 radiologically diagnosed RCC cases, 64.8% were managed conservatively, while 35.2% underwent surgical intervention. Headaches, visual field defects, and endocrine disturbances were more prevalent in surgically treated patients [6]. Conservative management is appropriate for asymptomatic or small RCCs, involving observation with serial MRI and hormonal evaluations as needed. Surgery is effective for visual dysfunction but may not significantly improve quality of life within the first postoperative year [7]. Recurrence risk factors include suprasellar location, inflammation, reactive squamous metaplasia, superinfection, and the use of a fat graft in the cyst cavity [8]. Diagnosing RCC can be challenging, particularly in distinguishing it from craniopharyngiomas, which share a similar embryological origin. An accurate differential diagnosis is essential. Despite advancements in diagnostic imaging, therapeutic strategies, and knowledge of disease progression, debates persist regarding the optimal management of RCCs. This review provides a historical overview of RCCs, explores their anatomical and embryological features, discusses developments in molecular genetics, clinical and radiological characteristics, differential diagnosis, and offers updated recommendations for follow-up and treatment.

## Historical background

Rathke’s pouch and cleft, prominent eponyms in medical literature, honor Martin Heinrich Rathke (1793–1860), a German anatomist celebrated for his pioneering contributions to embryology and comparative anatomy [9, 10]. In 1838, he hypothesized that the pituitary gland develops from a diverticulum of the embryonic stomodaeum, a theory later validated by other researchers in the 1870s [1].

### Heinrich rathke

**Fig. 1:**
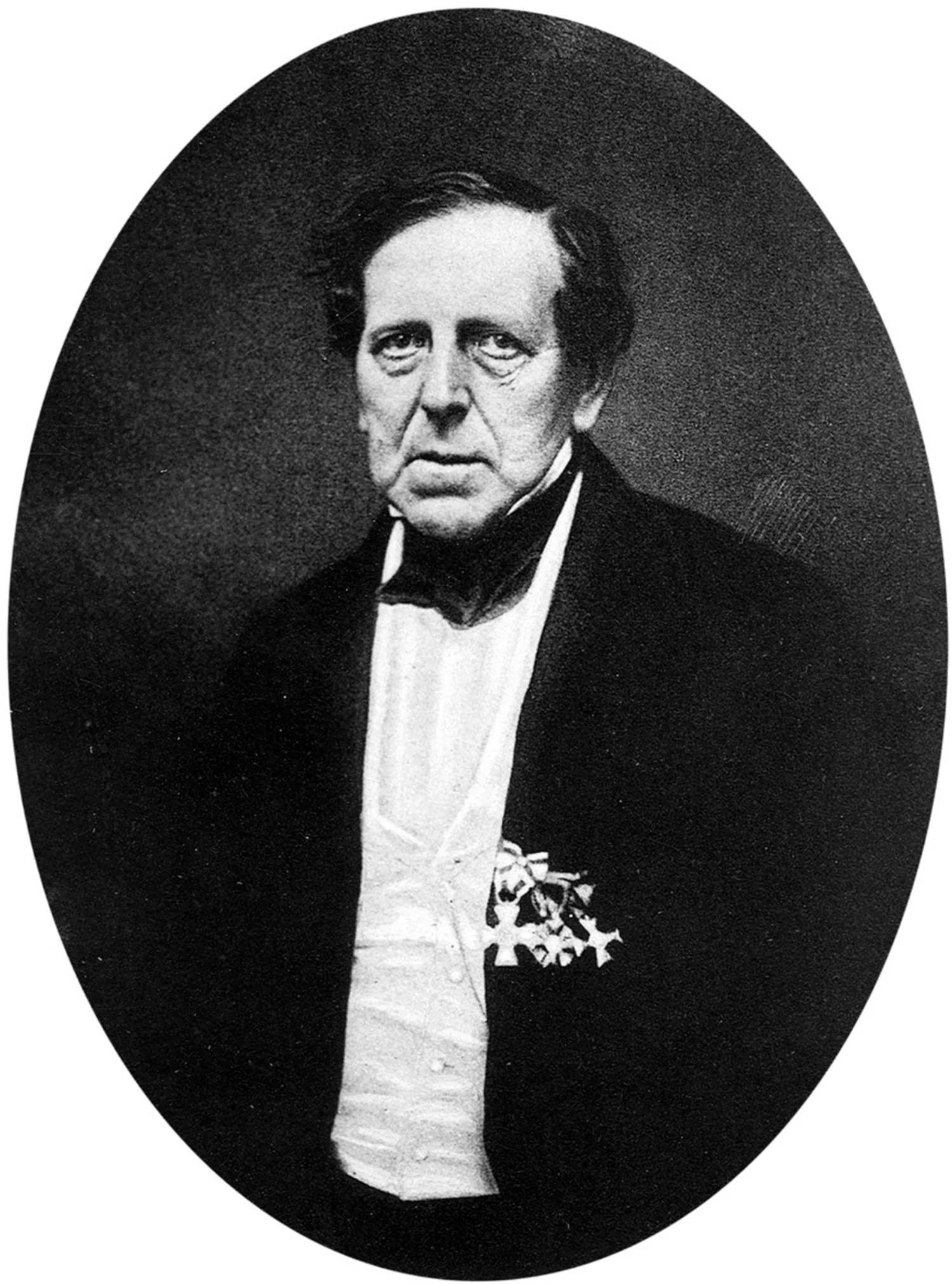
Martin Heinrich Rathke in 1850 (courtesy of the University of Tartu, Estonia)

Martin Heinrich Rathke was born on August 25, 1793, in Danzig, Prussia (now Gdansk, Poland) (Fig. 1), to a prosperous shipbuilder. After earning his MD in 1818 at Göttingen and Berlin, he returned to Danzig to practice medicine [10]. Pursuing zoological studies at Göttingen, he conducted original research during his student years [5]. In 1825, Rathke became chief physician at the city hospital, and from 1829 to 1835, he chaired the Physiology Department in Dorpat (now Tartu, Estonia). During this period, he conducted extensive research, focusing on marine zoology [1]. There, he completed the research he had begun in Danzig, concentrating on the historical formation and development of both humans and animals [11].

In 1832 and 1835, he traveled to the Crimea and Black Sea regions to better understand the fauna and collect zoological materials. In 1835, he succeeded Karl Ernst von Baer (1792–1876) as professor of anatomy and zoology at Albertus University (now Kaliningrad, Russia). Rathke undertook scientific excursions to Norway and Sweden in May 1839, then returned to Albertus University where he served as the rector from 1852 to 1853 and continued working until the end of his life. He died on September 15, 1860, in the same city [10].

After his investigations of the laws of formation in the animal body—amphibia and fishes in particular—he published several papers, among which the most significant were his monographs on the development of vertebrate animals’ genital system [12]. Rathke discovered the avian and mammalian structures that were homologous to the fish’s gill slits and described the embryonic origin of the pituitary gland [9]. He was elected a corresponding member of the Russian (1832), Göttingen (1851), Bavarian (1858), and French (1860) Academies of Science as well as the Royal Society of London (1855) [10, 12].

As one of the early pioneers of comparative embryology, Rathke authored nearly 125 works, including the classic Entwickelungsgeschichte der Wirbelthiere (Developmental History of Vertebrates), through his findings on homologous structures among various organisms [1, 13]. Gaining a wider recognition after the 1880s, Rathke’s name became associated with more than a dozen eponyms in medical terminology, although only a few (i.e., Rathke’s pouch, Rathke’s pocket and Rathke’s cleft) have been frequently cited in modern literature [2, 5, 9, 14–20].

### Rathke’s pouch, cleft, and cyst

In his 1838 paper entitled “Über die Entstehung der Glandula pituitaria” (“On the origin of the pituitary gland”), Rathke wrote the earliest description of the embryologic origin of the pituitary gland with the following words [21]:

I have long since noticed at a very early stage of the fetal life in several animals, particularly in mammals, a long time earlier when the palate is formed, at the very back of the oral cavity, below the base of the skull is a small, irregularly rounded indentation, that of the mouth’s mucous membrane and it was apparently a thin-walled bulge of the same. However, for a long time, I did not know how to interpret them, especially because I no longer found them in older embryos when I examined the oral cavity. At last, I became aware that this depression marks the first step in the formation of the brain’s appendage (the pituitary gland) (p. 482).

In the same paper, Rathke noted that this diverticulum later loses its connection with the epithelium of the digestive channel, uniting with the infundibulum, and finally forms the pituitary gland [21]. Historically, Rathke’s pouch was among the structures of problematic significance because its function and histological pathology remained unclear for decades. Embryologists studied and speculated on this formation for a long time. Two years after Rathke published his paper, Carl Bogislaus Reichert (1811–1883) suggested that the pituitary body was the residue of the anterior end of the notochord [22]. Later, Rathke revised his view, attributing pituitary formation to the mesoblast in front of the clinoid process.

In 1855, Robert Remak (1815–1865) also observed a similar process, which is “facing the base of the brain and still detectable at the time of the gill formation on the blind end of the glandular tube lining the pharynx” [23]. In 1860, Hubert Luschka (1820–1875) observed that “remnants of this part of the sella could be found anywhere along its pathway from the pharynx to the hypophysis” [24]. He first described the RCC as, “an epithelial area in the capsule of the pituitary gland that represents the oral mucosa” [25].

In 1869, Emil Dursy (1828–1878) noted that the notochord is united with Rathke’s pouch and takes part in forming the pituitary body [26]. In the following years, several researchers, including Wilhelm Müller (1832–1909), Alexander Wilhelm von Goette (1840–1922), and Géza Mihalkovics (1844–1899), also demonstrated that Rathke was correct in his first claim [27–29]. Defining the stages of formation of the anterior lobe of the pituitary gland, Mihalkovics (1875) pointed out that the cells forming Rathke’s pouch grow fast in the anterior wall [28, 30]. Luschka (1860) and Goldzieher (1913) documented the first RCC cases [4]. Historical milestones and experimental findings related to the pouch, cleft, and cyst named after Rathke are summarized in Table 1.

**Table 1:** Historical milestones and experimental findings related to Rathke’s pouch, cleft, and cyst

Year	Historical development
1838	Rathke wrote the earliest description of the embryologic origin of the pituitary gland [21]
1860	Luschka first described the RCC as “an epithelial area in the capsule of the human hypophysis resembling oral mucosa” [25]
1869	Dursy claimed that the notochord is united with Rathke’s pouch and takes part in the formation of the pituitary body [26]
1875	Mihalkovics noted that the cells creating Rathke’s pouch grow at varying speeds, with those in the anterior wall multiplying the most rapidly [28]
1913	Goldzieher described an early case of RCC in a 34-year-old man with diabetes insipidus [31]
1920	Duffy described an instance of a sizable adenoma in the anterior lobe of the pituitary, weighing 77 g that was connected to an incidental cyst of Rathke’s cleft. This cyst was lined with ciliated columnar epithelium and contained small tubular glands within its wall [32]
1926	Kiyono reported an instance of hypophyseal cachexia that resulted from a colloid cyst, which was lined with ciliated epithelium [33]
1927	Peet noted that the cysts originating from Rathke’s pouch form between the anterior and posterior lobes of the pituitary gland, primarily having an intrasellar origin [34]. Additionally, Worster-Drought, et al., described a condition of dyspituitarism linked to a pituitary cyst that emerges from Rathke’s cleft, leading to secondary lesions in both the hypothalamic region and the ventricles [35]
1928	Fulstow described an intrasellar cyst lined with stratified squamous epithelium with long papillary projections [36]
1929	Rasmussen noted that, in the pars intermedia of human hypophyses, which are generally normal, tall columnar cells with many fine cilia can sometimes be found in the epithelium lining the residual lumen and cysts [37]
1932	Bailey stated that tumors of the hypophyseal duct may be divided into three types: 1) mucoid epithelial cysts; 2) simple squamous epitheliomas, and 3) adamantinomas [38]. Susman found that 30.4% of the 230 hypophyses he had examined contained epithelial rests of an embryonic character [39]
1934	Frazier and Alpers documented the first known instance of successful surgical intervention for a RCC in English literature, detailing a biopsied cyst that was lined with ciliated epithelium [40]
1947	Baar described two instances of pituitary cachexia in children, both linked with substantial colloid cysts of Rathke’s cleft, resulting in pressure atrophy of the anterior hypophysis [41]
1948	Bayoumi described a RCC with clinical features of a chromophobe adenoma [42]
1949	In a patient without hypophyseal symptoms, Schermann and Barretto Netto discovered total obliteration of the hypophysis caused by a cyst that was lined with ciliated columnar epithelium [43]
1951	Shanklin categorized hypophyseal cysts into four types: 1) Cysts formed by the dilation of Rathke’s cleft. 2) Microfollicular cysts originating from the tubuloracemose glands. 3) Cysts in the hypophyseal stalk. 4) Cysts that are a consequence of the degeneration of adenomas or subsequent to infarcts in the hypophysis [44]
1957	Northfield published a paper on Rathke’s pouch tumors [45]
1959	Berry and Schlezinger reported a case of a 49-year-old woman with progressive loss of vision who was found to have a cyst protruding from the sella and compressing the optic nerves [46]
1965	Deneka presented a case report of a 58-year-old woman with a clinically apparent hypopituitarism caused by a Rathke’s pouch cyst [47]
1970	Shuangshoti suggested that the formation of cysts doesn't always stem from the Rathke pouch. Instead, neuroepithelium might be separated to create ependymal-lined tubules within the pituitary gland and surrounding the stalk [48]
1974	The advent of CT and MRI scans provide a better understanding of the surgical anatomy of Rathke’s cleft
1991	Voelker, et al., shared the clinical, radiographic, and pathological observations from 155 patients who were experiencing symptoms of a RCC [49]
1995	Graziani, et al., proposed the hypothesis of a common embryological origin of suprasellar neurenteric cysts, RCCs, and colloid cysts from endodermal remnants [50]
1999	Hayashi, et al., conducted MR and biomedical analysis of RCC contents [51]
2005	Aho, et al., reported the surgical results of 118 patients experiencing symptoms of a RCC [52]
2006	Sato, et al., proposed that the basal cells of a RCC undergo a transformation into a papillary type craniopharyngioma after squamous metaplasia [53]
2011	Chaiban, et al., defined a newly characterized distinct clinical entity regarding RCC apoplexy [54]
2019	Wong, et al., proposed the novel use of a bio-dissolvable stent to treat a recurrent RCC [55]

## Embryology, molecular biology, and histopathology

### Embryology

Oral ectoderm and neural ectoderm are two different embryonic ectodermal tissues and comprise the pituitary gland (hypophysis). The anterior pituitary develops from the oral ectoderm and encompass the anterior and intermediate lobes, whereas the posterior pituitary origins from the neural ectoderm (ventral diencephalon). The release of the stimulating mediators from the neighboring tissues and homeodomain transcription factors regulate pituitary cell differentiation and renewal (56). Mutual interactions of these two signaling components give rise to the primordial primary tissue, which is Rathke’s pouch. During the 3rd and 4th week of embryogenesis, Rathke’s pouch develops with the rostral evagination of the primitive upper oral cavity, forming the anterior pituitary (adenohypophysis) after losing its connection with the pharyngeum.

The cells covering the Rathke’s pouch anterior wall proliferate and stuff a high percentage of the pouch space to develop the pars tuberalis and pars distalis. The posterior wall is the origin site of the pars intermedia. Following these stages, cellular differentiation processes lead the formation of five distinct cell types to form the mature pituitary gland, which are lactotrophs, gonadotrophs, somatotrophs, thyrotrophs, and corticotrophs [56]. Yet, in different organisms, the cells covering the anterior pouch wall do not proliferate at a level to fill the Rathke’s pouch, which allows a remnant space between the Rathke’s cleft pars distalis and intermedia.

**Fig. 2:**
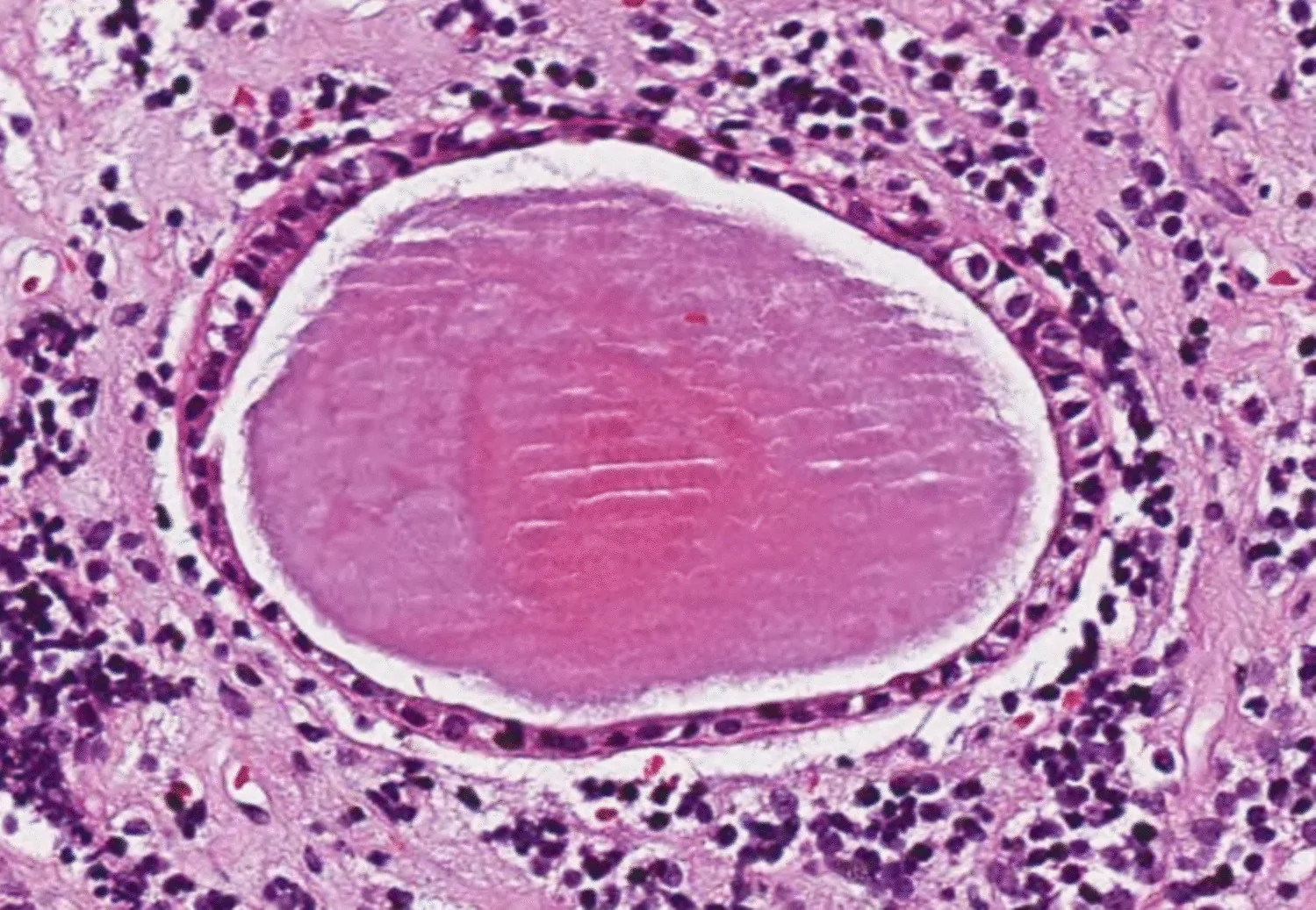
A Rathke’s remnant with characteristic cuboidal epithelium and eosinophilic cyst contents (H&E, × 500 original magnification)

**Fig. 3:**
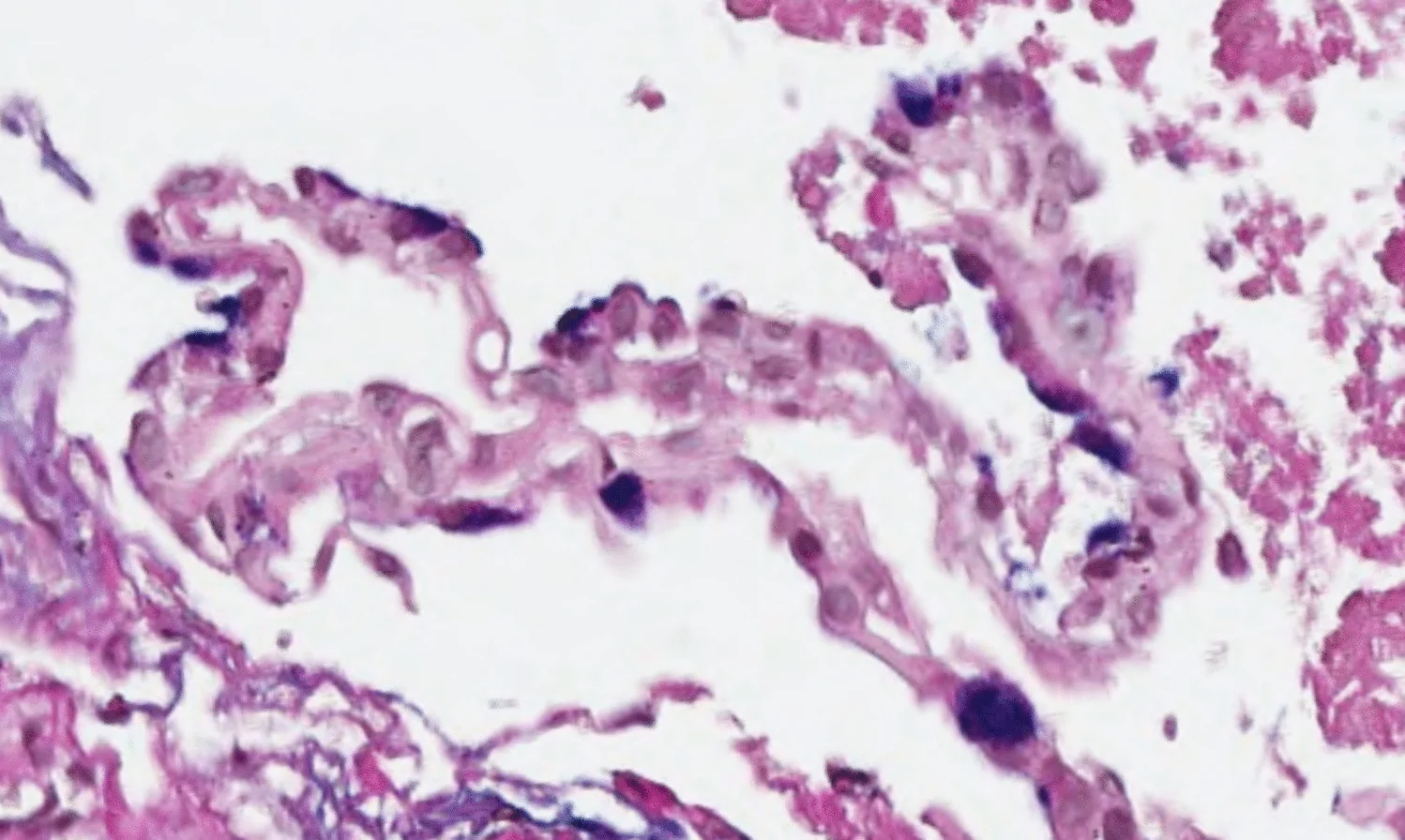
Scattered goblet cells between cuboidal overlying cyst epithelium (PAS-Alcian Blue, × 800 original magnification)

In humans, Rathke’s pouch remnants may lead to the development of the Rathke’s cyst, which contains mucus and is covered by cuboidal or columnar ciliated epithelia incapable of synthesizing trophic hormones [57] (Fig. 2). A possible cause of the RCC formation is the incomplete filling of the Rathke’s pouch during the 3rd or 4th week of embryonic development [58]. RCC epithelia, common with cilia, could be cuboidal, pseudostratified, or single-layered columnar, which morphologically resemble the neurenteric or endodermal colloid cyst epithelia, and scattered goblet cells secreting protein and cholesterol-containing mucin also frequently exist inside the cysts [58] (Fig. 3). Also, many RCCs show squamous differentiation with occasionally accompanying squamous cornified pearls. Residual adenohypophyseal tissue can be trapped at any anatomical site along the path followed by the Rathke’s pouch during the development [59–61].

The ectopic pituitary resides mainly in the sphenoid sinus and then in the immediate suprasellar region [62, 63]. These ectopic foci can be found incidentally or if they show hyperplastic or neoplastic alterations. The adenohypophysis deriving from the Rathke’s pouch is an epithelial gland made up of the pars distalis (anterior lobe), pars intermedia (intermediate lobe), and the pars tuberalis which is an epithelial extension wrapping the pituitary stalk’s infundibulum [64, 65]. The adenohypophysis comprises of acini which includes six differentially specialized hormone-secreting cells within a stroma rich in reticulin. Folliculostellate cells are stromal sustentacular cells immunoreactive for glial fibrillary acidic protein (GFAP) and S100 protein, which circumvent the acini. Uncommon follicular cells juxtaposing small follicles are presumed to originate from hormone-secretory cells following compression, degeneration, or trauma. The cellular differentiation and developmental events of adenohypophysis are controlled with highly selective transcription factors [66, 67]. Most lesions associated with the abnormal pituitary development localize in the sella turcica. Yet, ectopic adenomas of the pituitary can originate from Rathke’s cleft remnants, which may exist in the middle nasal meatus, parapharyngeal or suprasellar areas, sphenoid sinus, hypothalamus, third ventricle, clivus, or petrous temporal bone [68].

### Pathogenesis and molecular biology

The Rathke’s cleft and pituitary remnants may give rise to different pathological lesions because of their complex embryogenesis. In addition to adenomas, several other pathologies may develop, including benign cysts not limited to RCCs, such as epidermoid or arachnoid cysts, craniopharyngiomas, pituitary blastoma, pituitary or granular cell tumors, mixed gangliocytoma-adenoma, gangliocytoma, neurocytoma, and spindle cell oncocytoma. These diverse histopathological lesions may arise due to diverging molecular pathways. Below, these pathways regarding the continuing persistence of Rathke’s cleft remnants, RCCs, pituitary adenomas, and craniopharyngiomas will be discussed, which are relevant to the differential diagnoses of sellar pathologies as well as designing future molecular treatments.

#### Expressions of the cytokeratin and epithelial cell adhesion molecule (EpCAM)

Differential pathological diagnosis of RCCs and craniopharyngiomas is clinically significant, yet it may sometimes be challenging. Few research regarding the differential expression of cytokeratins (CKs) in RCCs versus craniopharyngiomas provided conflicting results. Early observations showed that 100% and 40% of the adamantinomatous and papillary craniopharyngiomas were positive for CK7, CK8, and CK14, respectively, where CK7 and CK8 were present in the ciliated cuboidal epithelia, and CK7 and CK14 were present in the metaplastic squamous cells of the RCCs [69]. Another pathological study analyzed cytokeratin expression in pars intermedia from the normal pituitary glands, RCCs, and craniopharyngiomas [70]. The control group and RCCs were positive for cytokeratin 2 and 8, yet craniopharyngiomas were negative for these [70].

Thus, it is conceivable to assume that RCCs and craniopharyngiomas arise from diverging cell types or different parts of the developing pouch. A RCC case with a small squamous metaplastic component dedifferentiated into a squamous papillary craniopharyngioma with a Ki67 index above 20% indicating high cellular proliferation, and notably, CK8 was positive in the initial RCC lesion, which was lost in the craniopharyngioma [71]. Although anecdotal, this exciting case supports the assumption that RCCs and craniopharyngiomas arise from differing cell lineages, and two cell types possibly present in the former RCCs provided a source for another pathology. Yet, it is still possible that the loss of CK8 expression is due to a cell dedifferentiation event. EpCAM (Epithelial Cell Adhesion Molecule or CD326) is an epithelial cell membrane type-I glycoprotein, highly expressed in versatile neoplasia [72]. An immunohistopathological and gene-expression study showed the absence of EpCAM in papillary cranyopharyngiomas and its presence in adamantinomatous craniopharyngiomas and RCCs, albeit at lower rates in the latter [72].

#### Mutation and expression of the β-catenin

Aberrant regulation of the Wnt/β-catenin signaling pathway leads to developmental abnormalities and oncogenesis [73]. In the ventral diencephalon, the pituitary organizer is a specific region that expresses Fgf8, Fgf10, and Bmp4, which stimulates the Rathke’s pouch development. A lack of Wnt expands the pituitary organizer and enlarges Rathke’s pouch [74]. Wnt signaling is classified into a β-catenin-mediated canonical or noncanonical pathway independent of β-catenin. Ventral diencephalon cells express nuclear β-catenin in the diencephalon [74]. A study investigated β-catenin mutations in 41 craniopharyngiomas and 10 RCCs. Mutations in β-catenin, causing nuclear accumulation of β-catenin protein, existed in 77% of craniopharyngiomas and entirely in the adamantinomatous subtype, but none of Rathke’s cysts had these mutations [73]. Therefore, aberrant nuclear β-catenin staining was proposed as the most valid parameter to distinguish cystic craniopharyngiomas from Rathke’s cysts [75]. Yet, some reports show that nuclear β-catenin expression is associated with aggressive Rathke’s cysts and their squamous dedifferentiation [76]. In addition, there are case reports of without β-catenin expression that later appeared in the craniopharyngioma cell nuclei [77]. These observations are compatible with the proposal that adamantinomatous craniopharyngiomas originate from ectopic Rathke’s pouch remnants, which share features with odontogenic tumors. In contrast, papillary craniopharyngiomas may arise from metaplastic dedifferentiation of the anterior pituitary epithelia [78].

#### Mutation and expression of BRAF

Most sellar or suprasellar papillary craniopharyngiomas have BRAF V600E mutations recognized by the VE1 antibody [79]. VE1 staining discriminates papillary carcinomas from adamantinomatous craniopharyngiomas expressing β-catenin but not having BRAF mutations [79]. Unexpectedly, VE1 was also shown to strongly stain the cilia of Rathke’s cyst epithelia [79]. However, this feature may not stem from the fact that papillary craniopharyngiomas and RCCs originate from the same cells, as BRAF mutations are not shown with gene sequencing in RCCs. This paradoxical observation may be attributed to the fact that the VE1 antibody strongly stains the cilia of bronchial and fallopian epithelia, sperm flagella, and specific cells of the epididymis and nasopharynx. Moreover, VE1 also strongly stains ependymal cilia and ependymomas with ciliated cells in their microlumina [79]. Here, it is presumable that the VE1 antibody, possessing a high affinity to ciliated structures in versatile cell types, stains RCCs not due to the BRAF mutations but rather because epithelial cells lining the RCC walls possess cilia. Hence, staining with antibodies that document a BRAF V600E mutation is not proof that these structures own these mutations and originate from the same cellular lineage as papillary craniopharyngiomas. Indeed, documenting a V600E mutation is even suggested as a marker for the differential diagnosis of craniopharyngiomas and RCCs [80, 81].

#### Likely role of female hormones

One of the notable features of RCCs is their preponderance in females by a 2:1 margin [49]. The pubertal presentation and female preponderance of RCCs indicate that female sex hormones may be involved in growth and pathogenesis [82]. The estrogen receptor is expressed at mRNA and protein levels in adenohypophysis, originating from Rathke’s pouch [83]. Hence, it is likely that estrogen may be involved in the pathogenesis of RCCs. Studies in older women with a RCC also give clues to the involvement of female hormones in RCC pathology. One study revealed that 69% of women developed these lesions during premenopause and 31% after menopause, supporting the likely propagative roles of female sex steroids [84]. Nonetheless, immunohistochemical analysis of 13 RCC specimens revealed a lack of estrogen receptor-α expression in the RCC-lining epithelia but its presence in the neighboring pituitary cells [80]. However, these findings do not rule out the role of female hormones due to several reasons. First, the selected antibody clones for immunohistochemical analyses do not bind receptor subtypes, and their binding epitopes may differ due to subtle polymorphisms or somatic mutations in these lesions. Second, sex steroids may also act via paracrine manner by crosstalk between Rathke cleft epithelia and nearby cells of the pituitary.

#### Leukemia inhibitory factor

The leukemia inhibitory factor (LIF) is a member of the IL-6 type cytokines, which determine hematopoietic, uterine, neuronal, and metabolic functions and development [57]. LIF and its binding sites in the human fetus arise early, 14 weeks after conception, and LIF inhibits the differentiation of embryonic stem cells and selectively the primitive ectoderm development. LIF expression was first demonstrated in human fetal pituitary tissue at mRNA and protein levels, mainly in corticotroph cells [85]. LIF treatment of cultured fetal pituitary cells could directly stimulate ACTH secretion, an activity shared by a related cytokine, Oncostatin M, which binds to a shared receptor [85]. LIF and LIF receptors are present in cells of the adenohypophysis, where they control the proliferation of pituitary cells and hormone synthesis [57]. In the pituitary, folliculostellate cells express LIF. Both fetal and adult pituitary express LIF and its receptors. In the pituitary, folliculostellate cells express LIF in 20% to 30% of cells producing ACTH and GH and 10% to 15% of cells that lack hormone synthesis or produce TSH, PRL, and gonadotropin, respectively [86]. In transgenic mice with forced LIF production driven by growth hormone gene promoter, three offsprings had decreased body weight and growth, robust anterior pituitary LIF expression, and cystic invaginations in the Rathke cleft’s anterior wall [57]. These cystic cavities were lined by cuboidal ciliated epithelia also strongly expressing LIF, focally S100 protein and cytokeratin, and did not stain with adenohypophysis hormones. LIF-overexpressing transgenic mice also exerted perturbed pituitary differentiation with expansion of corticotroph cells and simultaneous suppression of all other pituitary cell types. Lastly, a part of the same investigation revealed that all of the ten examined human RCC specimens expressed LIF [57]. A subsequent transgenic mice model created by forced LIF production, demonstrated the development of dwarfism with low IGF-1, hypogonadism with low FSH, Cushingoid features of thin skin and truncal obesity. Same as the previous transgenic model of pituitary LIF overproduction during development, corticotroph cell population showed expansion while somatroph and gonadotroph pituitary components were prominently hypoplastic. In similar, multiple RCC-resembling structures developed lined with ciliated epithelia [87].

#### PROP-1 in RCC development

PROP-1 (Prophet of Pit-1) gene encodes a homeodomain transcription factor protein, which is necessary for the expression of Pituitary-specific positive transcription factor-1 (Pit-1) [56, 82]. Human and mouse PROP-1 gene mutations indicate its crucial requirement for proper pituitary ontogenesis, which expression is limited to the developing pituitary as it stimulates undifferentiated precursor cell expansion in the Rathke pouch [56]. In humans, PROP-1 inactivation causes combined pituitary deficiency [88]. Even in the adult pituitary tissue, stem cells in the marginal zone express PROP-1 besides the other markers of embryonic stem cell including KLF4, OCT4, and SOX2 [89]. PROP-1 expression is observed before the transcription of most hormones while it is not detected at or beyond parturition. PROP-1 is particularly involved in the development, function, and differentiation of lactotrophs, thyrotrophs, somatotrophs, and gonadotrophs [82]. Gain of function gene mutations of PROP-1, which lead to aberrant constitutive expression of PROP-1 protein, delay the terminal dedifferentiation of gonadotrophs, induces pituitary adenomas, and causes the formation of RCCs [56].

#### SOX2 stem cells and the hippo signaling pathway

The definitive Rathke pouch has a central lumen lined by uncommitted stem cells expressing Sex-determining region Y (SRY) box-2 (SOX2) [89]. SOX-2 haploinsufficiency in humans causes hypopituitarism associated with a blockage of gonadotroph hormone synthesis. Mice with a conditional deletion of SOX-2 in Rathke pouch exert prominent hypoplasia of the pituitary anterior lobe, robustly reduced levels of the pituitary-specific transcription factor POU class-1 homeobox-1 (POU1F1), and strong defects in differentiation of thyrotroph and somatotroph cell lineages [90]. The stem cell features of SOX-2 expressing cell lineages were defined by their self-renewal and differentiation into pituitary cells with versatile hormone producing activities in organoid cultures. Several physiological conditions could activate stem cell population in the pituitary, albeit at limited levels and with a decreasing trend in later ages. The dormancy and activation of pituitary stem cells involve several signaling cascades, which are controlled by the Hippo pathway, Wnt pathway components, different types of cytokines, and their cross-talk [91]. Progeny of these SOX2 + cells may differentiate into three lineages, characterized by transcription factor expressions, which consist of TPIT, SF1, and Pit-1, respectively. The proliferation of SOX2 + cells is highly influenced by the Hippo signaling pathway, which involves YAP and TAZ proteins [91].

Yes-associated protein (YAP) and its paralog transcriptional coactivator with PDZ-binding motif (TAZ) have crucial functions in cell proliferation, differentiation, survival, and embryonic development. YAP and TAZ act as cytoplasmic and nuclear shuttle proteins which induce gene transcription to promote anchorage-independent proliferation and help cells to overcome anoikis and contact-inhibition-associated growth control. Hence, deregulation of their levels to reach supraphysiological amounts may provoke carcinogenesis [92]. Hippo protein acts as a tumor suppressor by suppressing protumorigenic stages due to YAP and TAZ activities, and acts apoptotic and antiproliferative. Hippo gene-inactivating mutations cause improper increases in organ size and oncogenesis. The Hippo signaling is crucial for proper development of the pituitary during the intrauterine development, and necessary to preserve and control SOX2 stem cells in adult pituitary [91]. YAP and TAZ are strongly expressed in nuclei of SOX2 stem cells in the embryonic Rathke pouch, postnatal anterior pituitary, in human pituitary tumors including adamantinomatous craniopharyngiomas and null-cell adenomas [91]. A constitutively active YAP form (S127A) in the hypothalamic primordium and Rathke’s pouch blocks cellular lineage commitment and maintains progenitor state, and causes the formation of RCC-resembling cysts having secretory multi-ciliated cells, which express acetylated α-tubulin and p63 [91].

#### Possible role of the Von-Hippel Lindau gene and protein

Von Hippel Lindau (VHL) protein involves in hypoxia signaling and its mutations cause an improper signal of hypoxia with subsequent activation of HIF-1α (Hypoxia Inducible Factor-1α). The first study regarding VHL protein expression in normal pituitary gland showed its presence in eosinophilic cells [93]. A succeeding study showed variable expression of VHL protein mainly in adenohypophysial cell cytoplasms and weak diffuse expression in the posterior pituitary lobe. Of note, most pituitary adenomas also stained positively with VHL with differing intensities and distributions, and the common localization of VHL protein in cell nuclei in poorly vascularized somatotroph adenomas was hypothetically linked to an inhibitory function of VHL in pituitary angiogenesis [94]. In this context, development of an aggressive pituitary macroadenoma in a patient with a family history of Von Hippel-Lindau Disease (VHLD) was attributed to the anti-angiogenic role of the native VHL protein in the pituitary gland [95]. Indeed, lesser expression of the native VHL protein in pituitary adenomas was found to highly associate with increased expression of the angiogenic VEGF protein and high rates of recurrence [96].

VHLD is a hereditary syndrome manifested with benign or malignant tumors and is caused by VHL tumor suppressor gene mutations [84]. VHLD is divided into 2 types according to the phenotype-genotype correlation [84]. Type 1 VHLD is caused by truncation or large deletion mutations producing an aberrant protein VHL having very low or absent activity. Type 2 VHLD develops due to missense mutations encoding a VHL protein variant with reduced yet not completely absent activity. Type 2 VHL may manifest with pheochromocytomas but not with other tumors. Huff et al. reported twin sisters with Type 2C VHLD and RCCs [84]. Both of these patients developed pheochromocytomas before 20 years of age. This interesting simultaneous occurrence of two different pathologies may indicate a novel mechanism of RCC pathogenesis. VHL regulates cilia maintenance, extracellular matrix interactions, cell polarity, development of renal and pancreatic cystic structures, and is expressed in all embryonic germ layers. The authors proposed an association with a malfunctioned VHL isoform and the development of cystic formations in the ectodermal Rathke pouch [84]. It may be presumed that aberrant VHL protein-associated hypoxic signaling caused co-occurrence of pheochromocytomas and RCC in twin patients with VHLD. Therefore, subtle mutations and/or polymorphisms of the VHL gene shall be analyzed in future studies regarding RCC formation.

#### Transcription factor ISL1

LIM proteins are homeodomain transcription factors possessing distinctive double-zinc finger motif domains with essential roles in pituitary cell differentiation and development [85]. Belonging to this family, Lhx3 and Lhx4 are critical to regulate the cell proliferation and differentiation in the Rathke’s pouch. Lhx2 is demanded for the genesis of the pituitary posterior lobe and infundibulum [85]. In humans, Lhx3 and Lhx4 mutations result in the combined pituitary hormone deficiency leading to the delayed or completely inhibited puberty [97]. Fuzzy Planar Cell Polarity Protein (FUZ) is obligatory for the normal ciliary organelle development through the retrograde recruitment of proteins mediating intraflagellar transport. Mice homozygously deleted of the FUZ gene entirely lack pituitary development. Paradoxically, the initial Rathke’s pouch develop normally in these mice, yet later undergoes apoptosis of its cellular content leading to hypoplasia co-occurring with a failure to express Lhx3 protein [98]. Considering the ciliated epithelia covering the RCC cyst walls, compelling interactions may take place regarding the LIM signaling pathways, involving ciliary organelle and pituitary development and pathologies related to the Rathke’s pouch aberrant development. Rathke’s pouch cells and the neighbouring ventral diencephalon also express another LIM homeodomain transcription factor, Islet-1 (ISL1) [85]. Immunohistochemistry analyses show ISL1 expression in the normal pituitary and in about 50% of the pituitary neuroendocrine tumors [99]. Pituitary stem cells as well as gonadotroph and thyrotroph cells produce ISL1 protein and lack of this expression results in hypopituitarism, apoptosis of stem cells, diminished gonadotroph and thyrotroph differentiation, and reduced body size [85]. Notably, conditional deletion of the ISL-1 results in multiple RCC development with 100% penetrance, which seems to associate with the deviant expression of the transcription factors FOXA and FOXJ1.

Aberrant production of these transcription factors link to a gene-expression pattern related to intracystic cilia formation. FOXA1, FOXJ1, and certain stem cell markers lack in craniopharyngiomas but are expressed in RCCs indicating their potential for pathological diagnosis of RCCs [85]. ISL1 expression among the pituitary stem cells induces thyrotroph and gonadotroph differentiation and the absence of ISL1, FOXA1 and FOXJ1 implicates the emergence of mucinous and oral epithelia cysts [85]. These cysts enlarge with age and their cells express the canonical RCC markers including certain cytokeratins, the acetylated form of tubulin and FOXJ1 [85]. In the mouse RCCs, FOXA1 appear as the earliest marker followed by an aberrant GATA3 localization in the cell nuclei accompanied with abnormal ectopic expression of the CDKN1A and FOXJ1 [85]. In the primordial pituitary, ISL1 may inhibit the expression of FOXA1, which regulates the alternate cell differentiation pathway partly through the FOXJ1 (85). Human RCCs express FOXA1 in a percentage about 94% which is absent in craniopharyngiomas. Lastly, sporadic synthesis of the SOX2 and SOX9 in the RCCs are parallel to developmental expression alterations in the ISL1-knockout mice [85].

### Histopathology

**Fig. 4:**
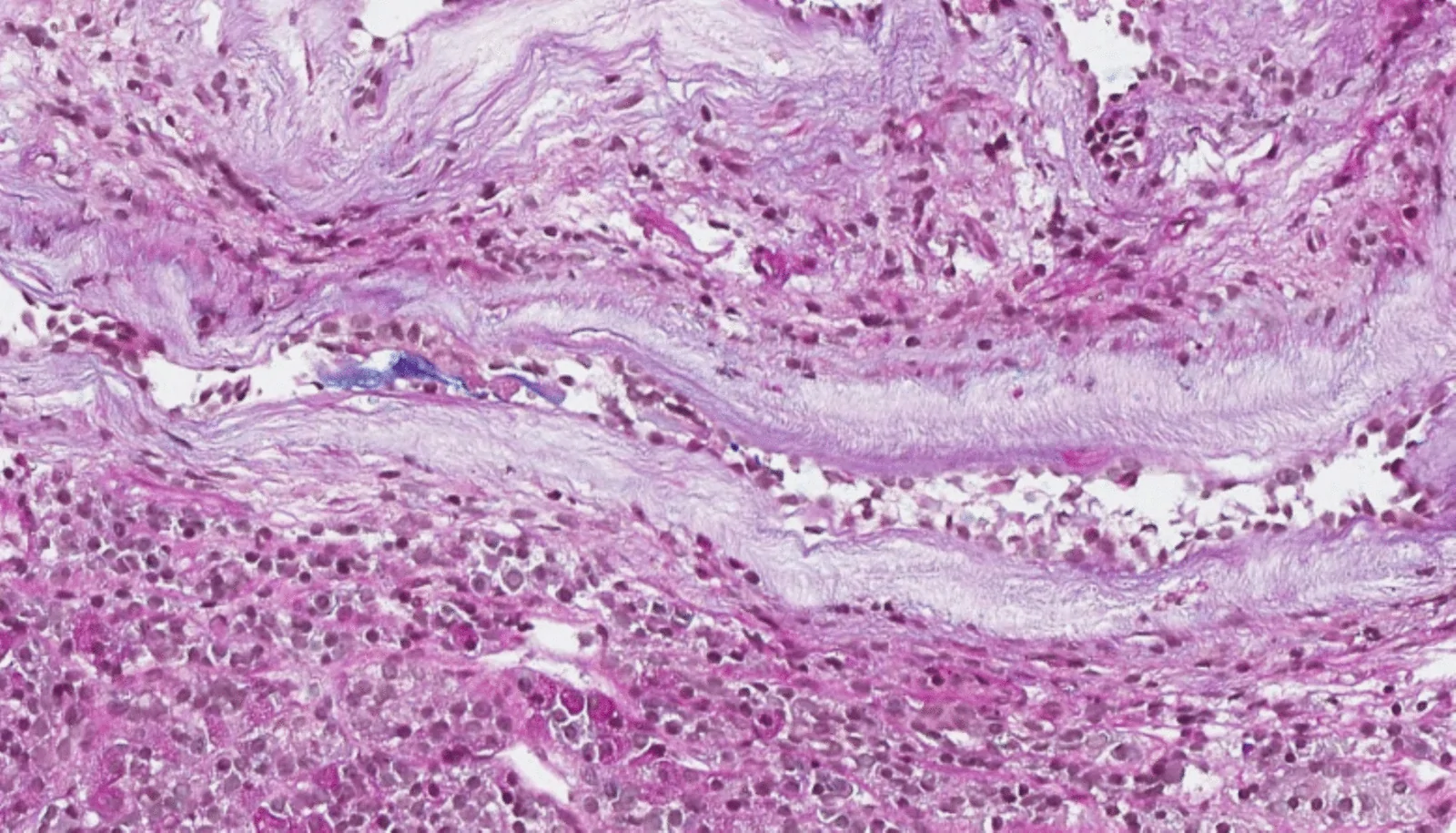
Partly exfoliated cuboidal epithelial cells of a Rathke’s cyst and adjacent anterior pituitary tissue (H&E, × 260 original magnification)

Epithelial cysts can be roughly classified in three categories according to origin: neuroectodermal (choroid plexus or ependymal), ectodermal (dermoid or epidermoid), and endodermal (Rathke’s cleft, neurenteric, and colloid). These cysts may demonstrate certain secondary changes including epithelial atrophy, squamous metaplasia, ulceration, and cyst rupture with inflammation, hemorrhage, and granuloma formation hindering precise classification. In such cases, immunohistochemistry helps further characterization, yet limited samples can only be descriptively defined as a benign epithelial cyst, where diagnosis and treatment decisions mainly rely on radiology to rule out intracranial and extracranial connections [100]. Small cysts covered by the cuboidal epithelium in the pituitary pars intermedia are Rathke’s pouch remnants which could give rise to a RCC. In lesser situations, these cysts may be found incidentally by a postmortem examination, yet when the cysts are larger in size, they can cause endocrine or mass effect-related to visual abnormalities. Histopathology reveals RCCs having a faintly cloudy mucinous content and a thin wall (Fig. 4). RCCs are mainly lined with columnar or cuboidal epithelium with or without cilia, and commonly include Goblet cells. Peptide-containing cells exist rarely in the RCC epithelium, and likewise, the columnar or cuboidal epithelia may seldom show a stratified and metaplastic squamous differentiation. Occasionally, microscopic cysts with similar morphologies may be present in pituitary adenomas [101]. RCCs may reside in both the sellar and suprasellar areas, and similar to colloid and endodermal cysts, ciliated pseudostratified columnar epithelium lines RCCs with varying numbers of the scattered Goblet cells. The diagnosis of these lesions is easy when the biopsy specimens include parts of the epithelial lining, yet the pathologist seldom receives the cyst content, rather ample material samples with eosinophilic and amorphous features. In suitable clinical conditions (i.e., when the neuroimaging is consistent with RCC), “amorphous proteinaceous material complying with RCC” can be provided as a definition of the pathological diagnosis. Here, it shall be also underlined that the microscopic detection of the entrapped Rathke’s cleft remnants is a normal finding for the pituitary gland. Thus, this condition is expectable if the pars intermedia is sampled during the resection with transsphenoidal approach and therefore, diagnostician shall consider whether the pathological diagnosis and the clinical features are consistent. Further, any transsphenoidal resection sample including RCCs or pituitary adenomas may contain miniscule components of the neurohypophysis, which may mimic neuroglial tumors, especially in the eye of a pathologist not much familiar with the regular histology of this anatomical region. Squamous metaplasia increases the likelihood of a papillary craniopharyngioma, yet the remnants of the respiratory epithelium are generally apparent in the latter. In necessary conditions, immunohistopathology for BRAF V600E can be made to confirm the pathological diagnosis, since the majority of craniopharyngiomas stain with the BRAF V600E antibodies, but not specifically the developmental cysts [102].

### Histochemistry and immunohistochemistry

**Fig. 5:**
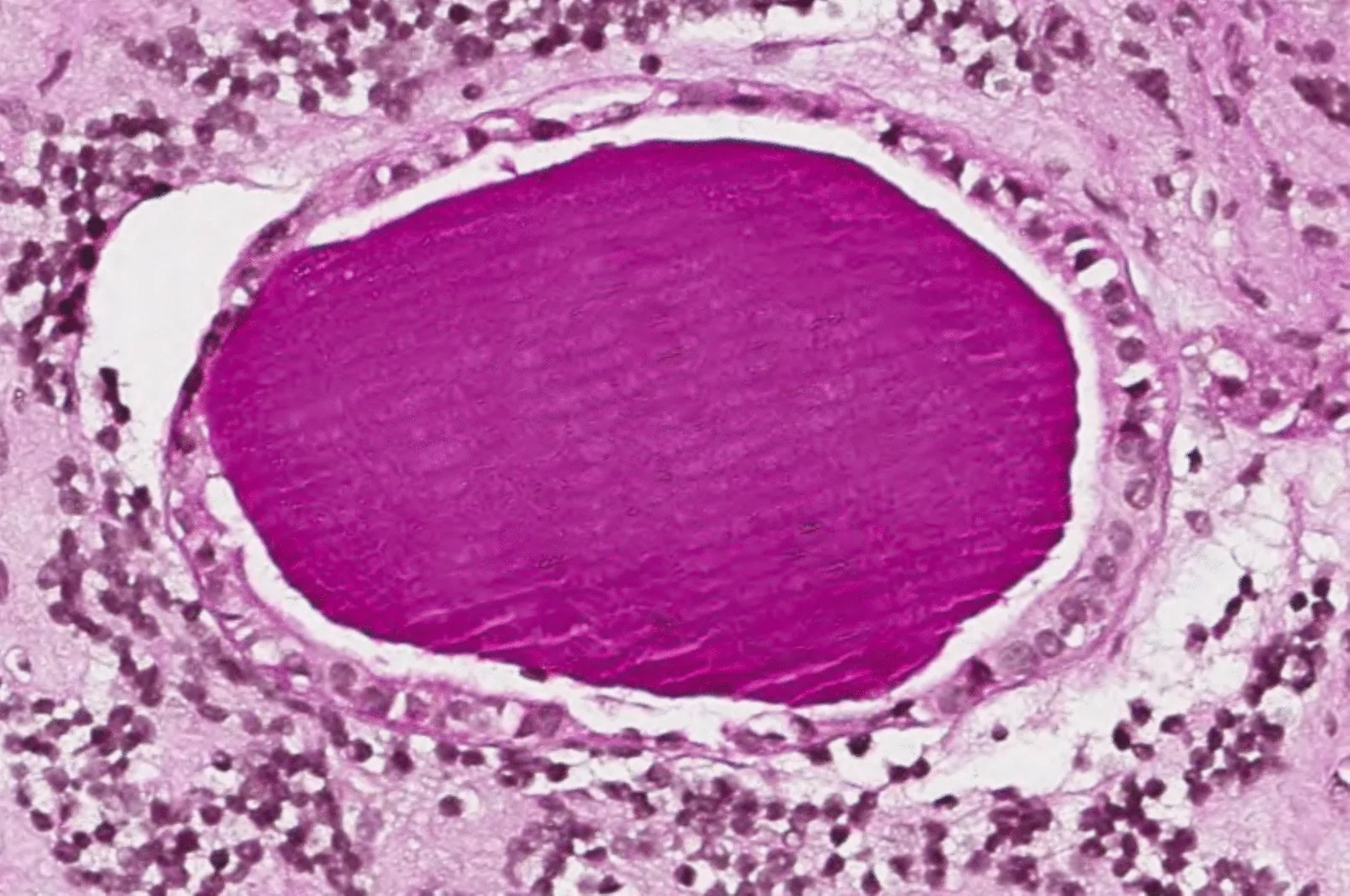
A Rathke’s cyst containing PAS + content (PAS, × 500 original magnification)

**Fig. 6:**
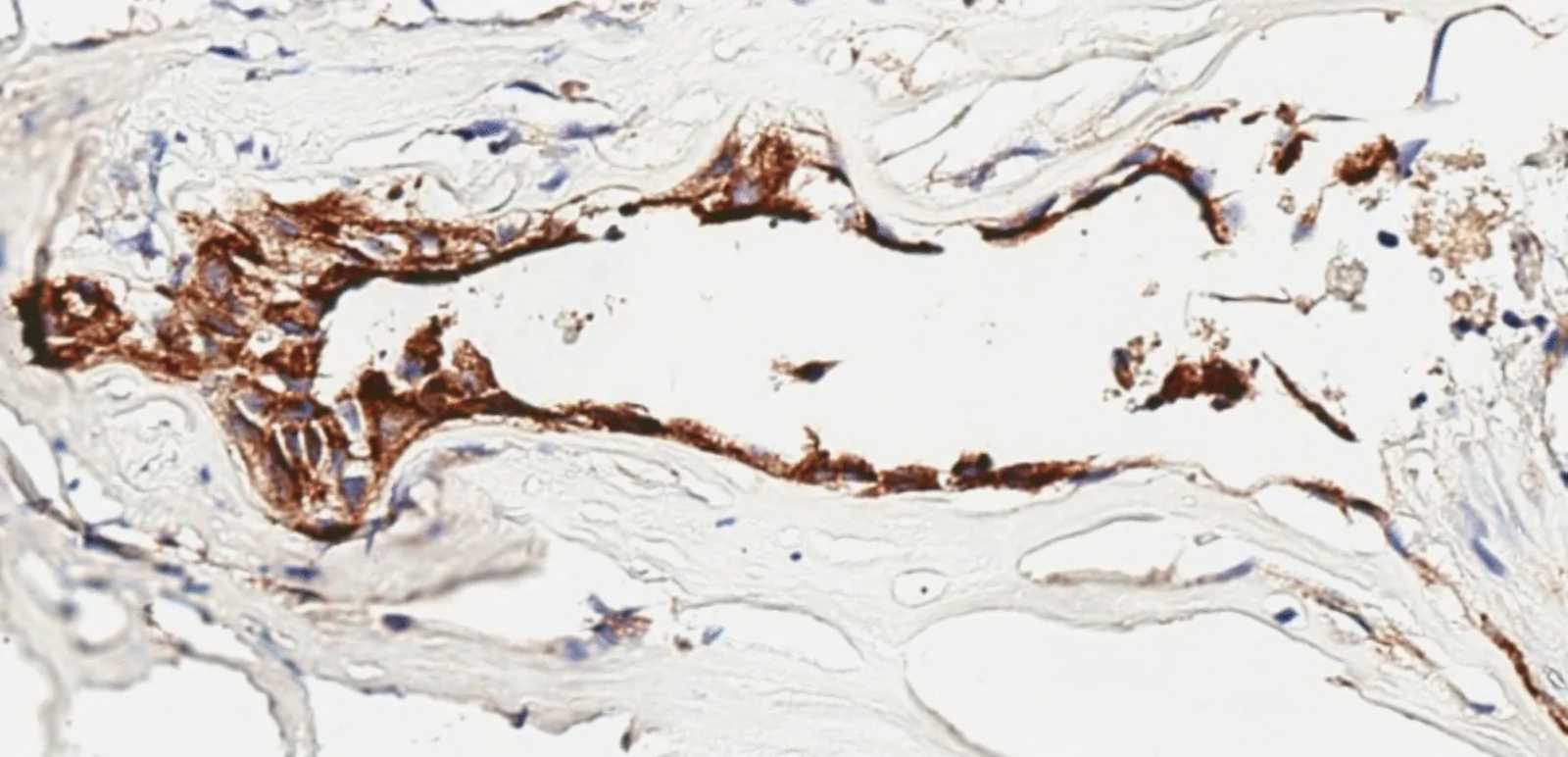
EMA-reactive cyst epithelium (EMA, biotinylated streptavidin complement, × 500 original magnification)

**Fig. 7:**
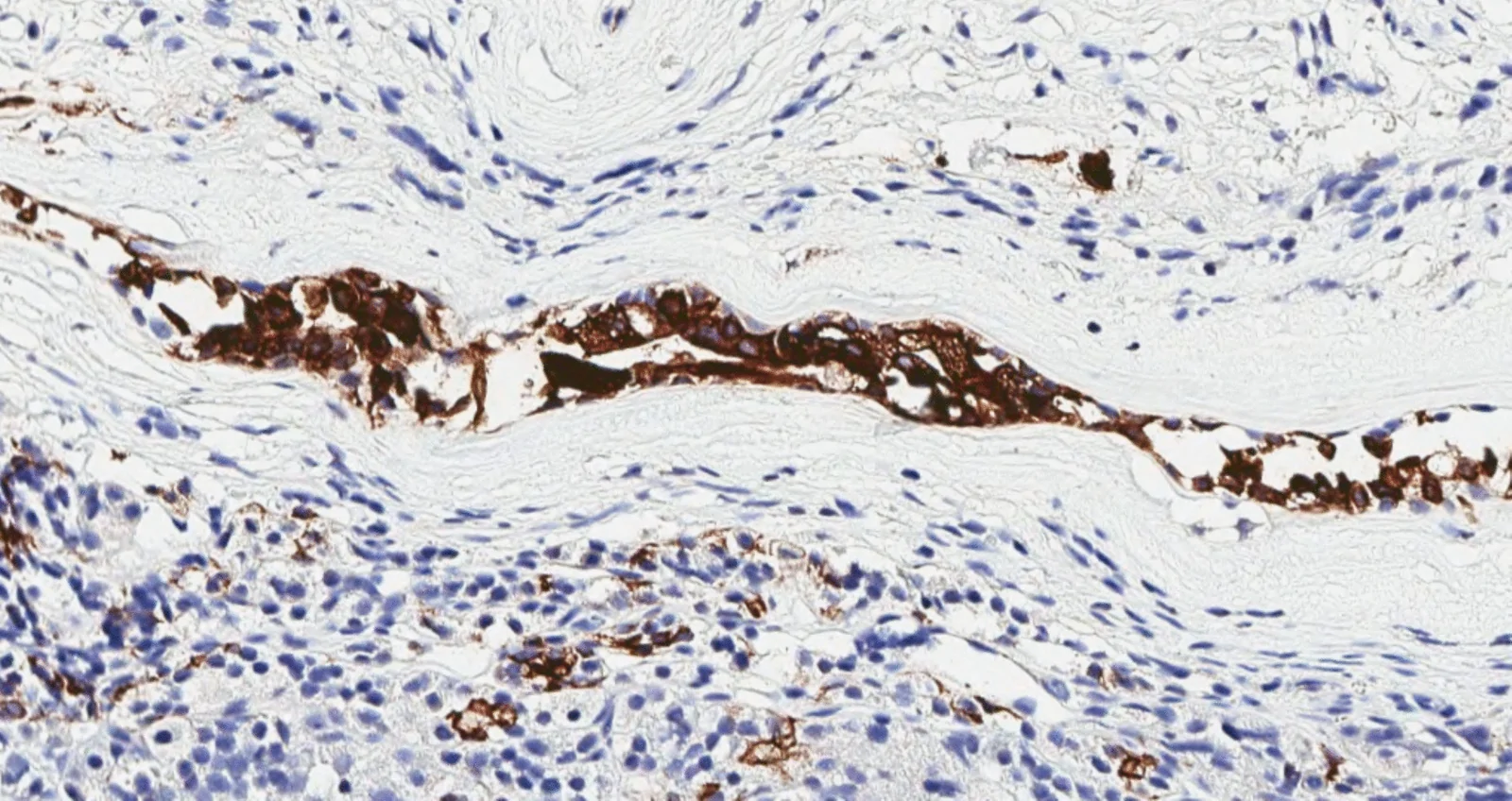
CK7-reactive cyst epithelium (CK7, biotinylated streptavidin complement, × 400 original magnification)

Mucin staining demonstrates the Goblet cells in the endodermal cysts including RCCs, colloid, and neurenteric cysts (Fig. 5). Immunohistochemistry of RCCs demonstrate the collagen IV staining of the subepithelial basement membranes, where the epithelia consistently express EMA (Fig. 6) and cytokeratins including the CK7 (Fig. 7), but generally not the CK20. RCCs stain inconsistently with CEA and they are negative for S100 protein, transthyretin, and GFAP [103–105]. RCCs with intense squamous metaplasia can mimic papillary craniopharyngiomas in microscopy, macroscopy, and radiology, yet as mentioned, BRAF V600E is positive in these lesions. Therefore, some authors stand with the proposal for a transition between these two pathologies because of the assumption of same precursor cells [106]. Nevertheless, in rare cases of uncertainty, a BRAF V600E stain can be applied as nearly all papillary craniopharyngiomas express this mutant protein whereas developmental cysts are uniformly negative [102].

## Clinical picture

Rathke cleft cysts are the most common masses with a rate of 28% to 42% among the nonpituitary sellar lesions of surgical series [107, 108]. The prevalence of RCCs as an incidental finding on MRI studies in young healthy subjects was 3.9% [109]. A large autopsy series reported an RCC rate of 11.3%, with 3.3% being larger than 2 mm, and both rates surpassing the rates of adenomas and hyperplasia [110]. The clinical picture for patients with an RCC is widely variable ranging from asymptomatic presentation to acute or chronic manifestations due to mass effects or panhypopituitarism. The high rate of autopsy series shows that most RCCs are asymptomatic and small. The widespread use of imaging has increased incidental RCC diagnoses, with most patients followed without interventions [6]. Asymptomatic RCCs increase in size at a rate of 5.3% during follow-up [111]. The optimal approach to these lesions is observation with periodic MRIs and hormonal analyses based on clinical status. Petersson, et al., reported no change in cyst size over 5 years in patients with baseline cysts of < 10 mm, and a follow-up of 5 years was recommended for these patients [112].

The symptomatology of RCCs may vary widely depending on the size, location, characteristics of the cyst fluid, and the presence of inflammation. We reviewed reports of surgical outcomes in patients with RCC from 1992 through 2022; presenting symptoms appear in Table 2. In most studies, the main symptom was headache, while endocrine and visual disturbances were two other common symptoms. The duration of symptoms before diagnosis varies widely with a mean of 14 months and a range of 3 days to 13 years in one study [113]. There is a female predominance among symptomatic RCCs (Table 2), which implies a role of female hormones, although another explanation was the earlier appearance of clinical manifestations in women secondary to hyperprolactinemia [112, 114].

**Table 2:** Demographics and the main symptomatology of the patients diagnosed with RCC that underwent surgery (studies from 1992 to 2022)

Author	Year	Number of Patients, n	Sex M:F, n	Main Presenting Symptom, n (%)	Preoperative Endocrinological Findings, n (%)	Preoperative Visual Disturbances, n (%)
Ross, et al. [115]	1992	43	10:33	Headache 19 (44%)	Hyperprolactinemia 8 (19%)	24 (56%)
El-Mahdy, et al. [116]	1998	28	12:16	Endocrine disturbances 14 (50%)	Amenorrhea 6 (21%) Hypopituitarism 4 (14%) Growth retardation 2 (7%) Diabetes insipidus 1 (4%)	25 (89%)
Benveniste, et al. [84]	2004	62	13:49	Headache 44 (71%)	Endocrine disturbances 34 (55%)	12 (19%)
Kim, et al. [113]	2004	53	25:28	Headache 34 (81%)	Endocrine disturbances 16 (30%)	36 (68%)
Aho, et al. [52]	2005	118	50:68	–-	Hypogonadism 56 (47%) Hyperprolactinemia 34 (29%) Hypocortisolemia 7 (6%)	58 (49%)
Frank, et al. [117]	2005	22	8:14	Endocrine disturbances 9 (41%)	Endocrine disturbances 14 (64%)	5 (22%)
Madhok, et al. [118]	2010	35	–-	Headache 26 (81%)	Endocrine disturbances 6 (18.8%)	–-
Wait, et al. [114]	2010	73	17:56	Headache 55 (75%)	Endocrine disturbances 36 (49%)	28 (38%)
Higgins, et al. [119]	2011	74	22:52	Headache 42 (57%)	Diabetes insipidus 10 (14%) Panhypopituitarism 10 (14%) Galactorrhea 9 (12%) Impotence 9 (12%)	18 (29%)
Jahangiri, et al. [120]	2011	161	36:125	Headache 55 (34%)	Hypopituitarism 54 (34%) Hyperprolactinemia 24 (15%) Diabetes insipidus 3 (2%)	25 (16%)
Lillehei, et al. [121]	2011	82	26:56	Headache 56 (68%)	Hypogonadism 28 (34%) Hyperprolactinemia 16 (20%) Hypothyroidism 10 (12%) Diabetes insipidus 7 (9%)	29 (35%)
Potts, et al. [122]	2011	151	32:119	Headache 62 (41%)	Endocrine disturbances 48 (32%)	20 (13%)
Xie, et al. [123]	2011	23	11:12	Headache 15 (65%)	Endocrine disturbances 6 (26%)	9 (39%)
Trifanescu, et al. [124]	2011	33	13:20	Headache 22 (67%)	Gonadotropin deficiency 20 (61%) ACTH deficiency 12 (36%) TSH deficiency 12 (36%) Diabetes insipidus 6 (18%)	19 (58%)
Zhong, et al. [125]	2012	45	21:24	Headache 29 (66%)	Endocrine disturbances 21 (47%)	44 (98%)
Solari, et al. [126]	2015	29	9:20	Headache 16 (55%)	Endocrine disturbances 15 (52%)	13 (45%)
Lin, et al. [127]	2018	100	27:73	Headache 66 (62.9%)	Hypogonadism 26 (24.7%) Hypopituitarism 15 (15%)	31 (29%)
Cabuk, et al. [128]	2019	84	17:67	Headache 56 (67%)	Endocrine disturbances 20 (24%)	17 (20%)
Wedemeyer, et al. [129]	2019	101	–-	Headache 52 (52.8%)	–-	23 (23%)
Marcus, et al. [130]	2020	61	20:41	Headache 25 (41%)	Anterior pituitary dysfunction 42 (69%)	22 (36%)
Arko, et al. [131]	2021	31	8:23	–-	–-	–-
Montaser, et al. [5]	2021	116	30:86	Headache 93 (80%)	Endocrine disturbances 40 (34%)	42 (36%)
Castle-Kirszbaum, et al. [7]	2022	38	10:28	Visual disturbances 21 (55%)	Hypopituitarism 10 (26%)	21 (55%)
Petersson, et al. [112]	2022	434	117:317	–-	Endocrine disturbances 58 (13%)	54 (12%)

The headache is generally frontal but may also be frontotemporal, occipital, or affecting the whole head [4, 49, 132]. It may be a dull or throbbing pain that is most commonly chronic [5], but may also be acute-onset, episodic, or progressive [4, 84, 106, 132]. In some patients, headache may be the only symptom or it may accompany other complaints [113, 116]. The duration of headache is highly variable, ranging from 1 week to 12 years with a median of 3 months in one report [84]. Compression of neighbouring neural structures, stretching of the sellar dura mater, and inflammation surrounding the cyst were among the factors suggested to cause the headache [126, 133].

A suprasellar location of RCCs was more commonly associated with headache than an intrasellar location [122], although this was not verified in all studies [125]. In many studies, the size of the lesion was not associated with headache, implying the presence of mechanisms other than compression or dural stretch [6, 120, 125, 132]. Nishioka, et al., reported an association between headache and hypopituitarism and signal hyper- or iso-intensity on T1-weighted images [132]. The mucous cyst contents may induce an inflammatory response causing headache and pituitary dysfunction [113, 132]. A correlation was reported between hyper- or iso-intensity on T1-weighted images, mucous content, and the presence of inflammation on histologic analysis [125, 132]. Contrary to these findings, a recent study reported no association between T1-weighted hyperintensity and headaches [6]. Another explanation for a headache independent of the size of the lesion might be increased intrasellar pressure [134].

Endocrine disturbances occur at a prevalence of 13% to 69% in patients with RCC (Table 2). Patients most commonly present with symptoms of hypogonadism, galactorrhea, and fatigue [106]. Nausea, vomiting, lethargy, hypotension, hyponatremia, polyuria, and polydipsia are among other signs and symptoms. Nevertheless, pituitary dysfunction might not be symptomatic in some patients and may be revealed only through testing [119]. Gonadotropin deficiency and hyperprolactinemia were generally the main hormone disturbances reported in the literature, while some authors reported growth hormone deficiency, central adrenal insufficiency, and central hypothyroidism as the most common [6, 7, 52, 84, 112, 113, 121]. El-Mahdy and Powell reported panhypopituitarism as the leading endocrine disturbance [116], but this condition was not supported by the majority of studies [84, 125]. Rarely, patients may present with symptoms of acute central adrenal insufficiency [135]. There are inconsistent reports regarding sex predominance for pituitary dysfunction caused by an RCC [112, 116].

Endocrine disturbances were not generally associated with the size and volume of the cyst [7, 132], but the location of RCCs was reported to be determinative over hormone deficiencies [126, 136]. Hyperprolactinemia and diabetes insipidus were more common among patients with a suprasellar RCC compared to those with an intrasuprasellar RCC, while hypopituitarism was more common in the latter [136], although not all studies supported these results [122]. Hyper- and iso-intensity on T1-weighted images associated with inflammation was more common in patients with pituitary dysfunction as stated above. Moreover, the presence of hyperprolactinemia was associated with low intensity, and diabetes insipidus with high intensity on T1-weighted images in the same report [132]. Inflammation was considered the reason for permanent pituitary dysfunction after surgery as the gland was already destructed [132]. On the other hand, as inflammation may cause episodic headaches, surgery was suggested for these patients to prevent loss of pituitary function [132, 137].

It is a known phenomenon that RCCs can decrease in size spontaneously. In one report, the regression rates during follow-up were 3.2% to 38% [6], and patients may experience a spontaneous relief of headaches [138]. This phenomenon was suggested to be associated with increased reabsorption over secretion of the cyst fluid, or rupture of or hemorrhage into the cyst [138]. There have been no predictive factors for spontaneous regression of the cyst [132], and there is lack of data about the rates of regrowth. Interestingly, there have been case reports describing significant involution of RCCs after glucocorticoid therapy, implying that the underlying inflammation had been suppressed [135, 139].

RCCs may lead to compression of the optic chiasm and cause visual field defects such as uni- or bilateral temporal hemianopsia or quadrantopsia and/or, more rarely, a decrease in visual acuity [112, 125]. The rate of visual disturbances is reported as 12.5% to 97% (Table 2). It was more common in patients with a suprasellar RCC and was associated with the size of the lesion [7, 122, 125, 132, 136].

RCCs can be complicated by intracystic hemorrhage or pituitary abscesses causing rare clinical manifestations. Patients with intracystic hemorrhage may present with severe, acute-onset headache, pituitary dysfunction, and visual deterioration, as well as nausea, vomiting, diplopia and signs of meningismus [140]. Hypogonadism was the most common hormonal deficit [140]. Apoplexy caused by an RCC was associated with a smaller lesion and more favorable endocrine and visual symptoms than that caused by a pituitary adenoma [54]. On the other hand, non-hemorrhagic rupture of the cyst may cause aseptic meningitis with severe headache and signs of meningeal irritation [121, 140]. The presence of an RCC poses a risk for developing a secondary pituitary abscess, although this is a rare complication [141]. Patients may present with sepsis along with a headache, pituitary hormone deficiencies, visual disturbances, and, more rarely, cranial neuropathies [141]. Gram-positive cocci are the most commonly isolated agents [141].

Other rare clinical manifestations might be caused by an atypical location or size of a RCC such as paralysis of oculomotor or abducens nerves, dizziness, epilepsy, and syncope [49, 142, 143]. Headache and diplopia were the main symptoms of intrasphenoidal RCCs [144], while compression of the brainstem was observed secondary to an RCC extending to the retroclival space [145]. Behavioral changes, alterations in mental state, and headache were reported in a patient with a huge RCC obliterating the third ventricle that caused hydrocephalus [146]. Rarely, a RCC may be associated with severe hyponatremia due to inappropriate secretion of antidiuretic hormone [147].

It may be challenging to differentiate RCCs from other sellar or parasellar lesions in terms of clinical presentation, radiology, and histology. Lesions that should be included in the differential diagnosis are craniopharyngiomas, cystic pituitary adenomas, arachnoid cysts, epidermoid cysts, epithelial cysts, dermoid cysts, and pituitary abscesses. Moreover, co-occurrence with other lesions may complicate the diagnostic process. A differential diagnosis is important as the treatment modality differs between diagnoses. Differentiation regarding histologic and radiologic investigations is reviewed under relevant sections in this article. The clinical aspect is discussed here.

The most difficult problem in diagnosing a RCC is differentiating it from a craniopharyngioma. Remnants of Rathke’s pouch commonly form small cysts. Although this condition may be seen occasionally in children, it is most common in adults. RCCs with extensive squamous metaplasia can mimic papillary craniopharyngiomas macroscopically, radiologically, and microscopically. However, some authors support the concept of a transition between these two entities because they derive from the same precursor [106]. Radiologic and pathologic investigations may be misleading [112, 113, 148], and some reports note that patients undergoing surgery for a recurrent RCC were eventually diagnosed with a craniopharyngioma [113, 121, 125]. The development of an adamantinomatous craniopharyngioma has been reported after the removal of a RCC [77]. This phenomenon may represent the development of a neoplasm from a cyst but might also reflect the misdiagnosis of the original lesion or a rare co-occurrence of the two lesions [113].

In clinical terms, patients diagnosed with a craniopharyngioma had a higher rate of visual disturbances and neurologic and psychiatric complaints when compared to those with an RCC [106]. The improvement of pituitary dysfunction after surgery was less likely in patients with a craniopharyngioma [106]. A recent study reported that the rates of headache and visual and endocrine disturbances were similar between patients diagnosed with a large RCC and a cystic craniopharyngioma [149]. Patients diagnosed with RCC were older than those with a craniopharyngioma [149].

The other diagnostic challenge could be a cystic pituitary adenoma. Both RCCs and pituitary adenomas can cause anterior pituitary dysfunction. Almistehi, et al., noted that the rate of pituitary hormone deficiencies depends not only on the size of the lesion but also on the histologic subtype [150]. They reported that multiple hormone deficiencies are associated with RCCs in comparison to prolactinomas [150]. RCCs have anatomic proximity to the posterior pituitary and may cause diabetes insipidus through compression or inflammation of the neurohypophysis [132]. Rarely, pituitary adenomas may coexist with RCCs and further complicate the diagnostic process [151]. Tavakol, et al., investigated the predictive factors of cystic sellar lesions and reported that the presence of symptoms associated with hyperprolactinemia and obesity predicted a cystic pituitary adenoma rather than an RCC [152]. The association between obesity and the presence of a pituitary adenoma was demonstrated in a recent population-based study [153].

The clinical course of RCCs can be complicated by the occurrence of sellar abscesses, as mentioned above. However, sellar abscesses might be encountered in a primary setting without an underlying RCC and may confound the diagnosis [154]. Asthenia was the most common symptom, followed by endocrine and visual disturbances and headache. Fever and leucocytosis were less common findings [154]. Arachnoid cysts may also cause headache, pituitary dysfunction, and visual problems, and should be considered during the diagnostic process [106, 151]. RCCs cause multiple hormone deficiencies more frequently than do arachnoid cysts [106]. Other cystic lesions that may pose a difficulty on histologic and radiologic terms are epithelial, dermoid, and epidermoid cysts [155]. Rarely, mature cystic teratomas with a sellar location have been misdiagnosed as RCCs on the basis of clinical and radiologic evaluation [156]. Neurologic symptoms and visual problems were more common among these patients. The diagnosis of these teratomas was established after pathologic confirmation [156]. A sellar xanthogranuloma is another rare tumor that may confound the diagnosis of an RCC [157]. These masses may occur secondary to an RCC as well as other sellar lesions. The clinical presentation may be similar to a RCC but panhypopituitarism is more common [157].

## Radiology

The initial CT and MRI findings of RCCs were not reported until the 1980s [158–163]. The first report was "symptomatic RCC demonstrated on CT" by Byrd et al. [158]. Since then, numerous radiological findings with CT and different sequences of MRI have been reported to disclose the secrets of RCC [132, 163–168]. Now, MRI is the gold standard in the diagnosis of a RCC with some specific findings such as intracystic nodules [169, 170].

### Rathke’s cleft cyst classification

The size of the lesion and evidence of any symptom are the basis for classifying RCCs, but they are mostly classified as symptomatic or asymptomatic [171]. Generally, RCCs smaller than 10 mm are asymptomatic and those larger than 12 mm are symptomatic. Micro-RCCs, accepted as anatomical variations, are seen more frequently with high-field MRI scanners [171]. Headaches, vision loss, visual field abnormalities, and pituitary endocrine dysfunction are typical symptoms of symptomatic RCCs [172]. Meningitis and pituitary abscesses are uncommon findings [173, 174].

### General radiologic findings

**Fig. 8:**
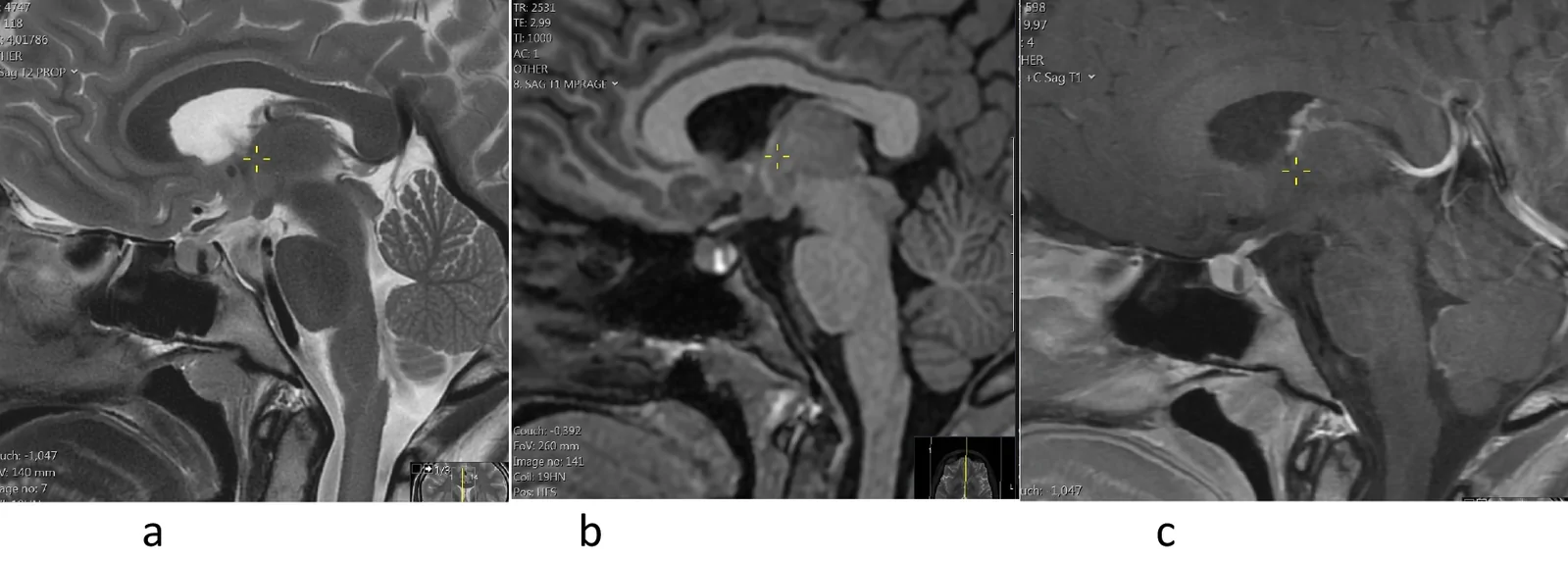
Sagittal T2- (**a**), T1- (**b**), and contrast-enhanced sagittal T1- (**c**) weighted images show the characteristic location of an RCC

**Fig. 9:**
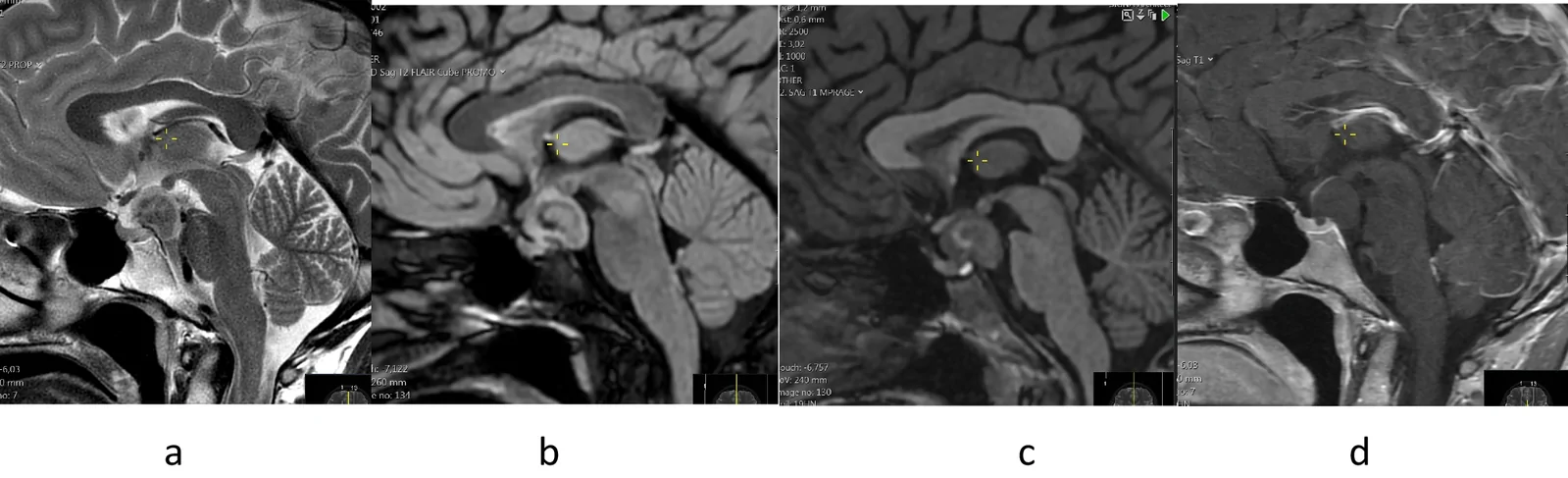
Ectopic RCC. Magnetic resonance images depict a nonenhancing suprasellar mass with an intracystic nodule that displays hypointensity on sagittal Flair and T2-weighted images (**a**, **b**) and iso- to slight hyperintensity on the sagittal T1-weighted image (**c**). There is no enhancement on the sagittal T1-weighted image after contrast administration (**d**). The lesion is located just dorsal to the pituitary stalk, with a small extension into the pituitary gland, and occupies the preopontine cistern

RCCs are mostly midline lesions, usually occupy the sella turcica, and often extend into the suprasellar region. When they are located in the intrasellar region and smaller than 10 mm, the typical site is between the neurohypophysis and adenohypophysis (Fig. 8). Exclusively suprasellar RCCs are very rare. RCCs located within the suprasellar region are typically seen on the superior aspect of the pituitary gland. However, more often than not, they exhibit some degree of extension into the intrasellar region, primarily involving the pituitary gland. Intrasellar RCCs are usually oval while RCCs extending into both intrasellar and suprasellar regions are either oval or dumbbell shaped with contraction at the diaphragma sella. Rarely, RCCs can be located outside the midline and sellar region, strikingly, even in the clivus [175]. An ectopic location is not uncommon (Fig. 9) [145]. The wall of an RCC is very thin, and all RCCs have smooth, lesional walls showing no invasion into the surrounding structures. The cyst wall does not enhance after the administration of contrast [176].

#### Signal intensity

The components of an RCC determines the signal intensity on MRI [167, 177]. An RCC may contain serous or mucoid fluid, with varying concentrations of cellular debris or constituents such as cholesterol particles, proteins, or mucopolysaccharides. As a result of differences in the concentration of cholesterol, protein, and blood products, each of which may provide a distinct signal, the MRI signal of RCCs is varied on both T1- and T2-weighted sequences. Crucially, the signal intensity of RCCs tends to resemble that of the neurohypophysis more closely than the adenohypophysis, mainly because of its high protein content. Consequently, differentiating an RCC from the large posterior pituitary lobe can pose challenges. However, this diagnostic confusion is unlikely when the pituitary gland experiences significant displacement due to the RCC.

#### Computed tomography features

RCCs appear as a well-defined, often non-calcified cystic sellar masses on CT scans, with or without a suprasellar extension [178]. The cyst contents affect the density of RCCs on CTs. Although they can be iso-or hyperdense, they are typically hypodense [178], and septations can be found in complex cysts. CT scans outperform MRI in showing neighboring bone remodeling [179]. Although the cyst wall may enhance in complicated cases, RCCs are normally non-enhancing. CT cannot provide a conclusive diagnosis on its own; thus, it is important to take into account the findings of MRI and biochemical, pathological, and clinical data.

#### MRI features of asymptomatic RCCs

**Fig. 10:**
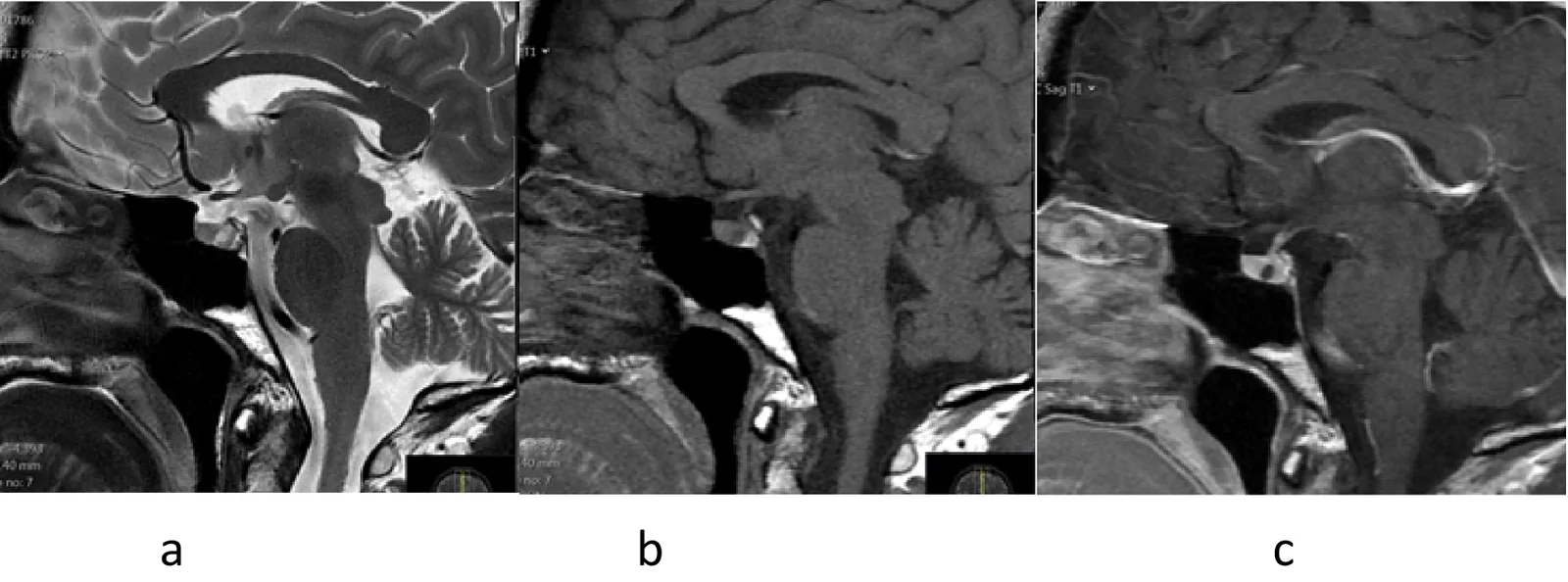
Serous RCC sagittal T2- (**a**), T1- (**b**), and contrast-enhanced sagittal T1- (**c**) weighted images show the characteristic shape and location of an RCC with a CSF-like signal

**Fig. 11:**
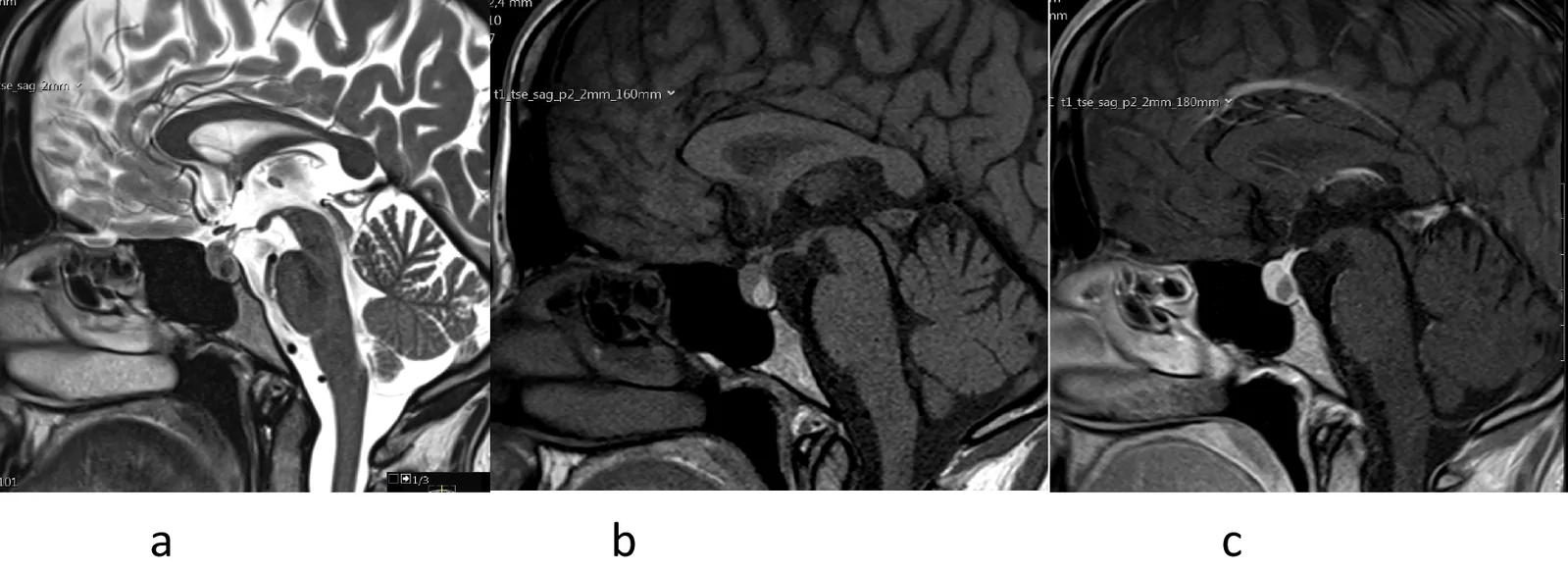
Hyperproteinic RCC. The sagittal T2-weighted image (**a**) demostrates a homogeneous hypointense mass located on the midline between the adenohypophysis and neurohypophysis. The sagittal T1-weighted image (**b**) shows the hyperintense signal of the lesion. The contrast-enhanced sagittal T1-weighted image (**c**) shows no enhancement of the lesion

RCCs smaller than 10 mm in size are generally asymptomatic, and almost all asymptomatic RCCs are discovered incidentally on imaging studies done for other reasons. The MRI appearance depends on the components of the cyst. Serous or mucoid fluid with varying concentrations of cellular debris or particles of cholesterol, proteins, or mucopolysaccharides may be present [51, 177]. When the cyst is thought to be serous, the signal is uniformly hyperintense on T2-weighted images and homogenously hypointense on T1-weighted images (Fig. 10) [51, 177]. The signal changes in accordance with the predominant cystic component if the cyst also contains mucoids, cellular detritus, or proteins. High protein levels reduce the T1 and T2 relaxation times, which affects the intensities on these images and causes the RCC to appear hyperintense on T1- and hypointense on T2-weighted images (Fig. 11) [51].

**Fig. 12:**
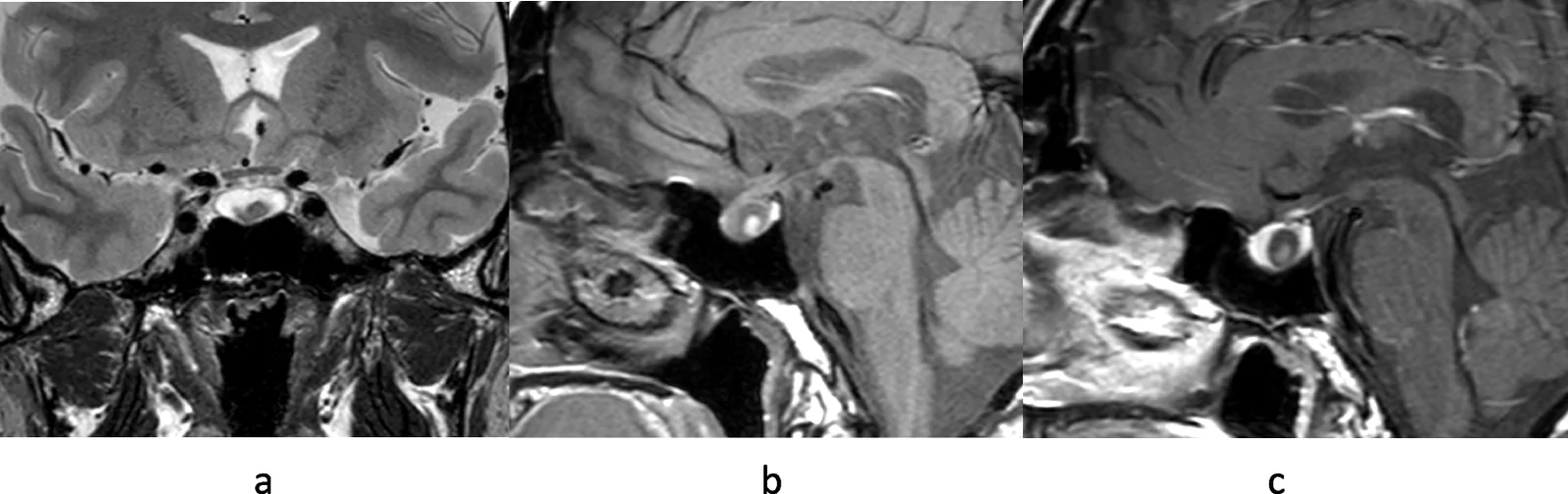
The case of a 43-year-old male with a surgically confirmed RCC with an intracystic nodule. Magnetic resonance images demonstrate an RCC with a nodule in the the cyst that displays low signal intensity on the coronal T2-weighted image (**a**) and high signal intensity on the sagittal T1-weighted image (**b**). The cyst and intracystic nodule show no enhancement on the sagittal contrast-enhanced T1-weighted image (**c**)

**Fig. 13:**
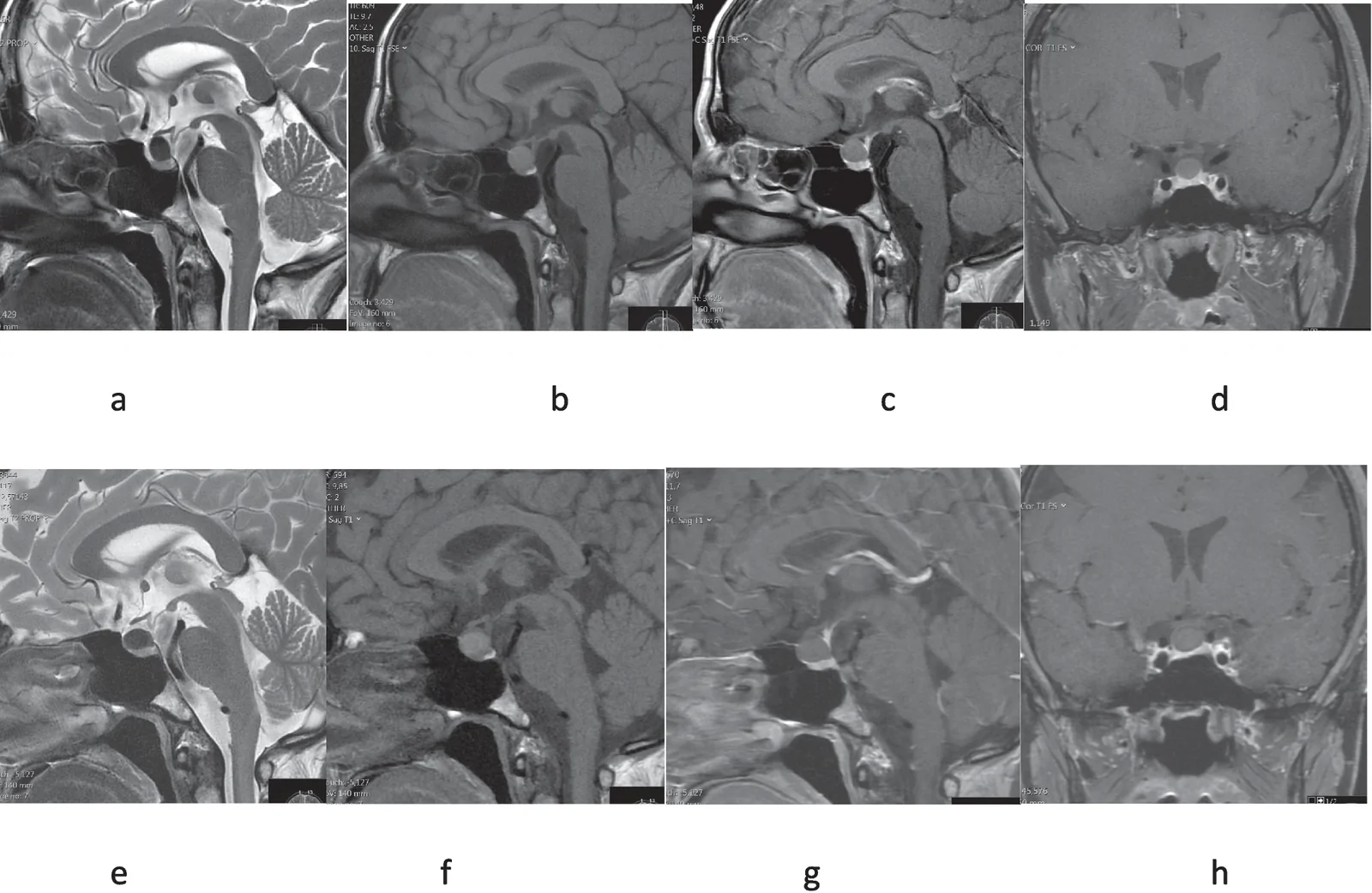
An RCC with an intracystic nodule. Magnetic resonance images show an RCC with a large nodule in the cyst that displays low signal intensity on the sagittal T2-weighted image (**a**) and slightly high signal intensity on the sagittal T1-weighted image (**b**). The cyst and intracystic nodule show no enhancement on the sagittal and coronal contrast-enhanced T1- weighted images (**c**, **d**). Magnetic resonance images taken 4 years after the initial MRI show a change in the position of the intracsytic nodule (**e**–**h**). This finding is diagnostic of an RCC

**Fig. 14:**
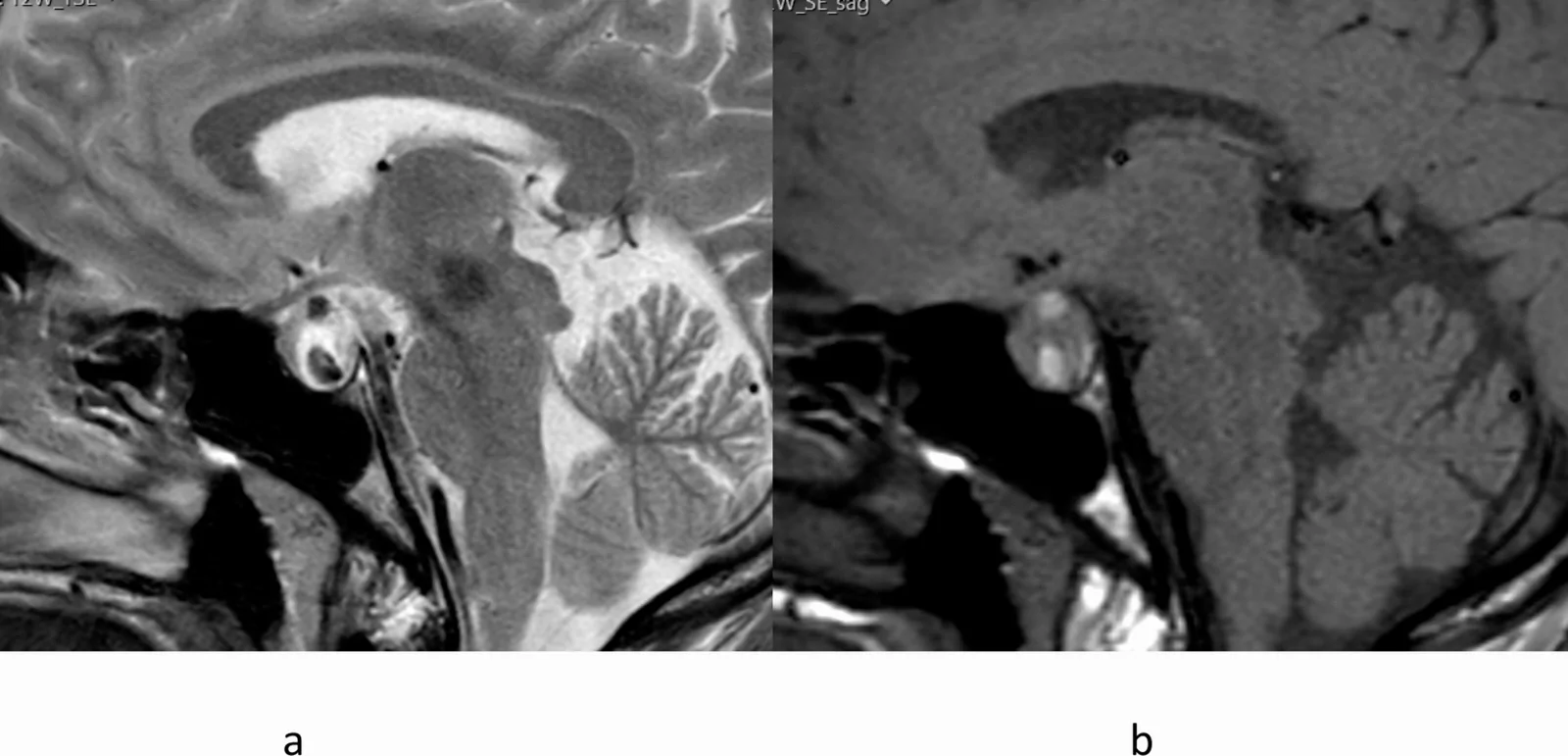
The case of a 34-year-old female with a surgically verified RCC with multiple intracystic nodules. Magnetic resonance images demonstrate an RCC with two nodules in the cyst that display a low signal intensity on the sagittal T2-weighted image (**a**) and high signal intensity on the sagittal T1-weighted image (**b**)

RCCs may contain intracystic nodules, which are reported in different percentages by different authors to range from 15 to 75%, and their presence within the cyst leads to the diagnosis of an RCC independently from clinic parameters (Fig. 12) [169, 170]. The main components of these nodules are protein, mucin, and cholesterol. Most of the nodules float freely within the cyst, and their position can change according to the patient's position (Fig. 13). A few nodules can adhere to the cyst wall. The signal of these nodules is isointense to hyperintense on T1-weighted and marked hypointense on T2-weighted images. They can be large, occupying almost the whole cyst, or multiple (Fig. 14). They do not enhance.

The wall of an RCC is very thin. Asymptomatic RCCs do not enhance after the administration of contrast material, although an enhancing rim of the displaced and compressed pituitary gland is present in approximately half of cases, thus mimicking wall enhancement. Contrast-enhanced dynamic studies may help prevent the confusion of interpretation between the normal pituitary tissue and cyst wall enhancement [165].

#### MRI features of symptomatic and complicated RCCs

RCCs that cause symptoms are infrequent. If they do appear, symptoms are either caused by the mass effect or a consequence of complications [180]. Headache, endocrine dysfunction, and vision loss are among the main symptoms linked to RCCs in large populations [4, 124, 180]. According to reports, 35% to 50% of individuals undergoing surgical intervention experience vision loss, which can include losses in both visual acuity and visual fields [132, 181, 182].

The most frequent endocrinological abnormalities linked to RCCs are hyperprolactinemia and growth hormone insufficiency, followed by hypocortisolemia and hypogonadism [182]. Diabetes insipidus is a documented presenting symptom in some RCC patients [183, 184]. Complications related to RCCs may include primary inflammation, infection, hemorrhage, apoplexy, and rupture [141, 185].

On CT scans, RCCs do not enhance after the introduction of contrast material. Inflammation of the cyst wall appears on MRI as rim enhancement after the introduction of gadolinium contrast. This inflammatory process might be linked to an intermittent foreign-body reaction incited by mucinous contents; hence, it is more frequently observed on T1-weighted scans. Inflammation of the cyst wall may escalate mucus secretion, subsequently leading to augmented cyst volume. Additionally, this inflammation can extend towards the adjacent anterior pituitary tissue, leading to secondary hypophysitis marked by deficiencies in gonadotropin, ACTH, TSH, and/or GH, alongside hyperprolactinemia. Instances of cyst infection and bacterial meningitis primarily manifest in mucinous RCCs.

The concept of shared venous drainage between the sellar contents and the sphenoid sinus has been postulated as a potential mechanism for secondary infection. Intracystic hemorrhage tends to appear in patients with underlying coagulation disorders. This occurrence is typified by the presence of a fluid–fluid level that is best discerned on sagittal and axial MRI views. The detection of a fluid–fluid level on T2-weighted images serves as a distinctive hallmark of a hemorrhagic event.

Clinically, apoplexy caused by an RCC is fairly uncommon. Its characteristics can be either hemorrhagic or non-hemorrhagic. The term "Rathke’s cleft cyst apoplexy" has been used for patients with abrupt-onset symptoms such as acute severe headache, meningismus, visual disturbance, oculomotor nerve palsies, pituitary function impairment, and imaging evidence of intracystic bleeding. The mechanism of this phenomenon is not well understood. The distinctive intracystic nodule of an RCC might be challenging to differentiate from the acute bleeding observed in pituitary apoplexy.

## Differential diagnosis

The primary differential diagnoses for RCC typically revolve around three other pathologies: craniopharyngiomas, cystic pituitary adenomas, and arachnoid cysts. The most prevalent MRI characteristics of asymptomatic RCCs include no discernible sellar change, a distinct midline intrasellar location, and close proximity to the posterior lobe. Additionally, the presence of T2-hypointense intracystic nodules and the absence of cyst wall enhancement detected on MRI often contribute to the diagnostic determination for most RCC cases [176]. It is crucial to differentiate RCCs from other cystic lesions in the pituitary to ensure appropriate treatment planning.

### Pituitary cystic adenoma

**Fig. 15:**
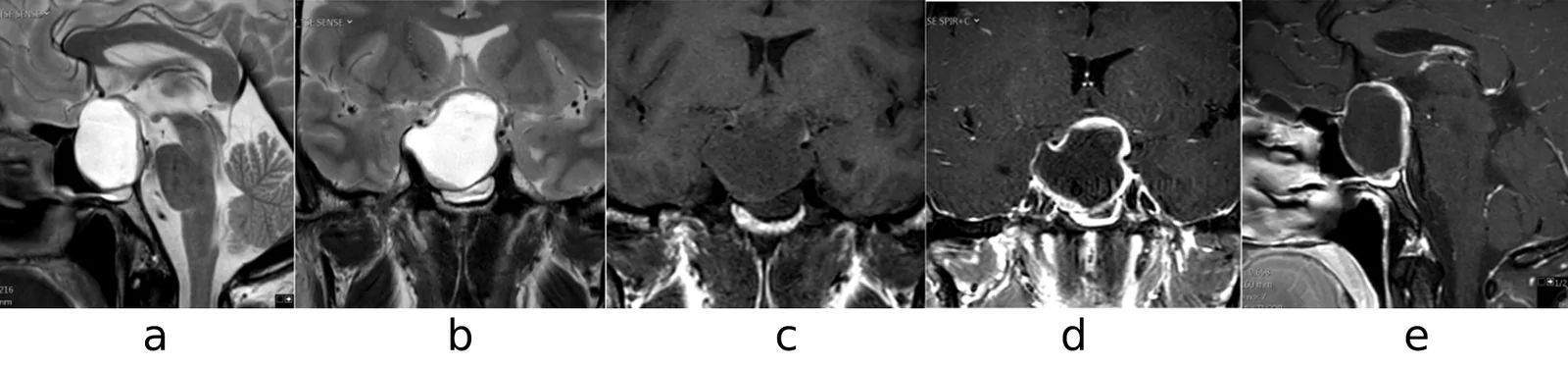
The case of a 43-year-old male with a surgically verified cystic pituitary adenoma. The sagittal and coronal T2-weighted images (**a**, **b**) and coronal T1-weighted image (**c**) show an intrasellar cystic mass extending into the suprasellar region. The cystic lesion reveals a thick wall on the contrast-enhanced coronal and sagittal T1-weighted images (**d**, **e**), which indicates a cystic adenoma of the pituitary rather than an RCC

On MRI, pituitary adenomas and RCCs exhibit substantial differences. The diagnosis of a cystic pituitary adenoma is greatly supported by the presence of specific MRI features, including a fluid–fluid level, a hypointense rim apparent on T2-weighted images, an off-midline position, evidence of septation, displacement of the pituitary stalk, and prominent wall enhancement (Fig. 15) [176, 186]. Notably, the likelihood of an intracystic nodule being present is significantly higher in RCCs compared to pituitary adenomas. When the normal pituitary gland surrounding an RCC enhances, there is the possibility of mimicking wall enhancement. Rapid enhancement assessment with dynamic MR imaging can help distinguish normal pituitary tissue from enhancement of the cyst wall.

### Craniopharyngioma

**Fig. 16:**
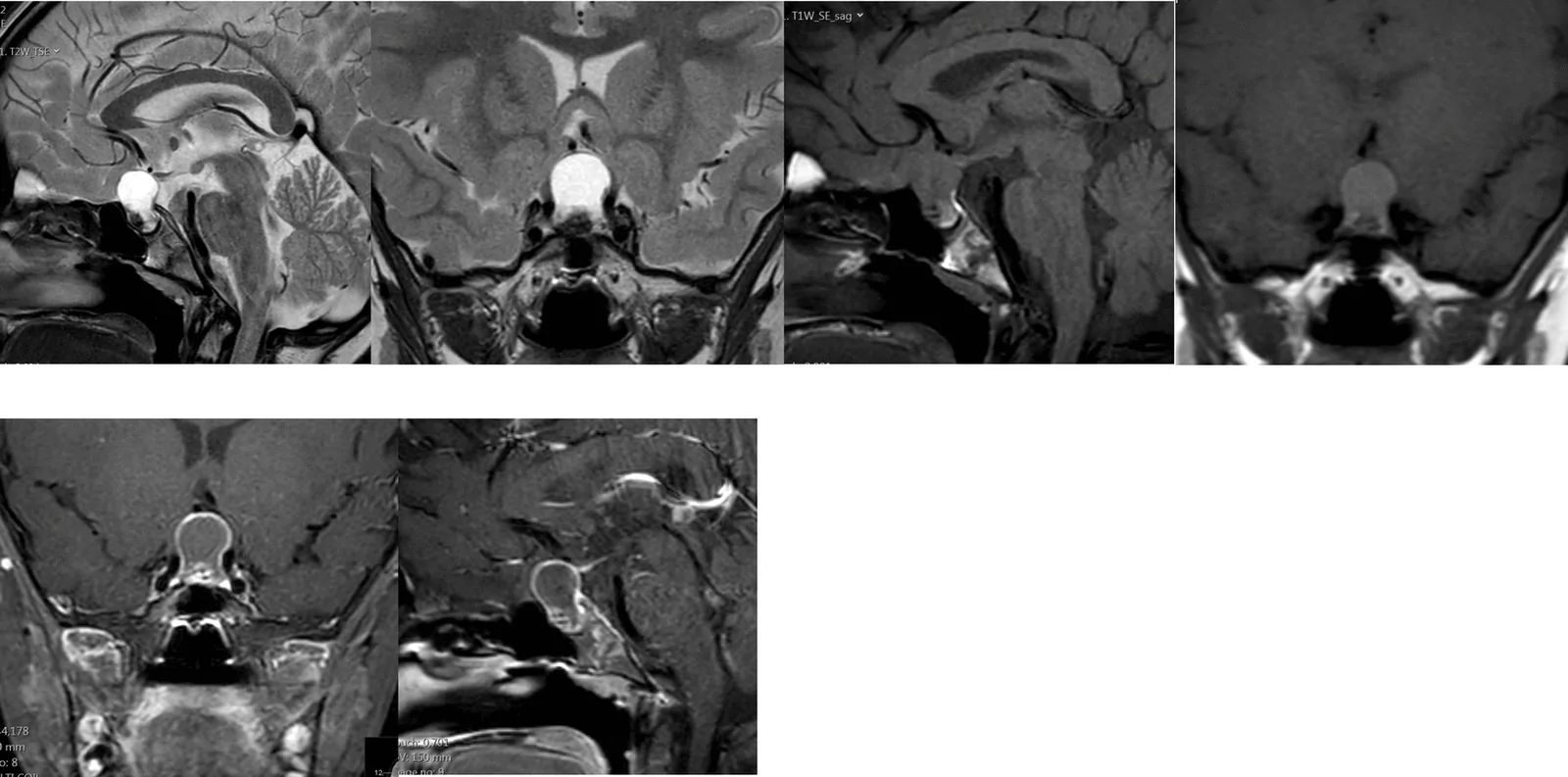
The case of a 10-year-old female with a surgically verified cystic craniopharygioma. Sagittal and coronal images of T2- and T1-weighted images (**a**-**d**) reveal a large and well-delineated sellar cystic lesion with a suprasellar extension. The lesion is hyperintense on a T2-weighted image and iso- to slightly hyperintense on the T1-weighted image. The lesion has a nodule in the cyst that displays low signal intensity on the sagittal and coronal T2-weighted images (**a**, **b**). The lesion shows wall enhancement on the sagittal and coronal contrast-enhanced T1-weighted images (**e**, **f**). Note that the nodule also shows contrast enhancement

Distinguishing between RCC and a cystic craniopharyngioma can be challenging based solely on MRI signal intensity. However, the presence of a thick, irregular enhancing wall (2 mm or more) displaying a reticular [187] or nodular pattern could indicate a solid component, which would favor the diagnosis of a craniopharyngioma (Fig. 16). While calcifications are a distinct hallmark of craniopharyngiomas, they are less frequently observed in purely cystic lesions [188]. These calcifications manifest as small hypointense nodules on T2-weighted, gradient echo T2-weighted and susceptibility-weighted images. Nevertheless, CT is the preferred method to detect calcifications. Larger lesions, those exceeding 2 cm, have been associated with a higher likelihood of being craniopharyngiomas, and a more pronounced superior lobulation is often indicative of craniopharyngiomas as well. Furthermore, compression of the third ventricle tends to be more commonly observed in patients with craniopharyngiomas. Psychiatric symptoms, along with the presence of calcifications or solid components on imaging, are more strongly linked with craniopharyngiomas.

### Arachnoid cysts

**Fig. 17:**
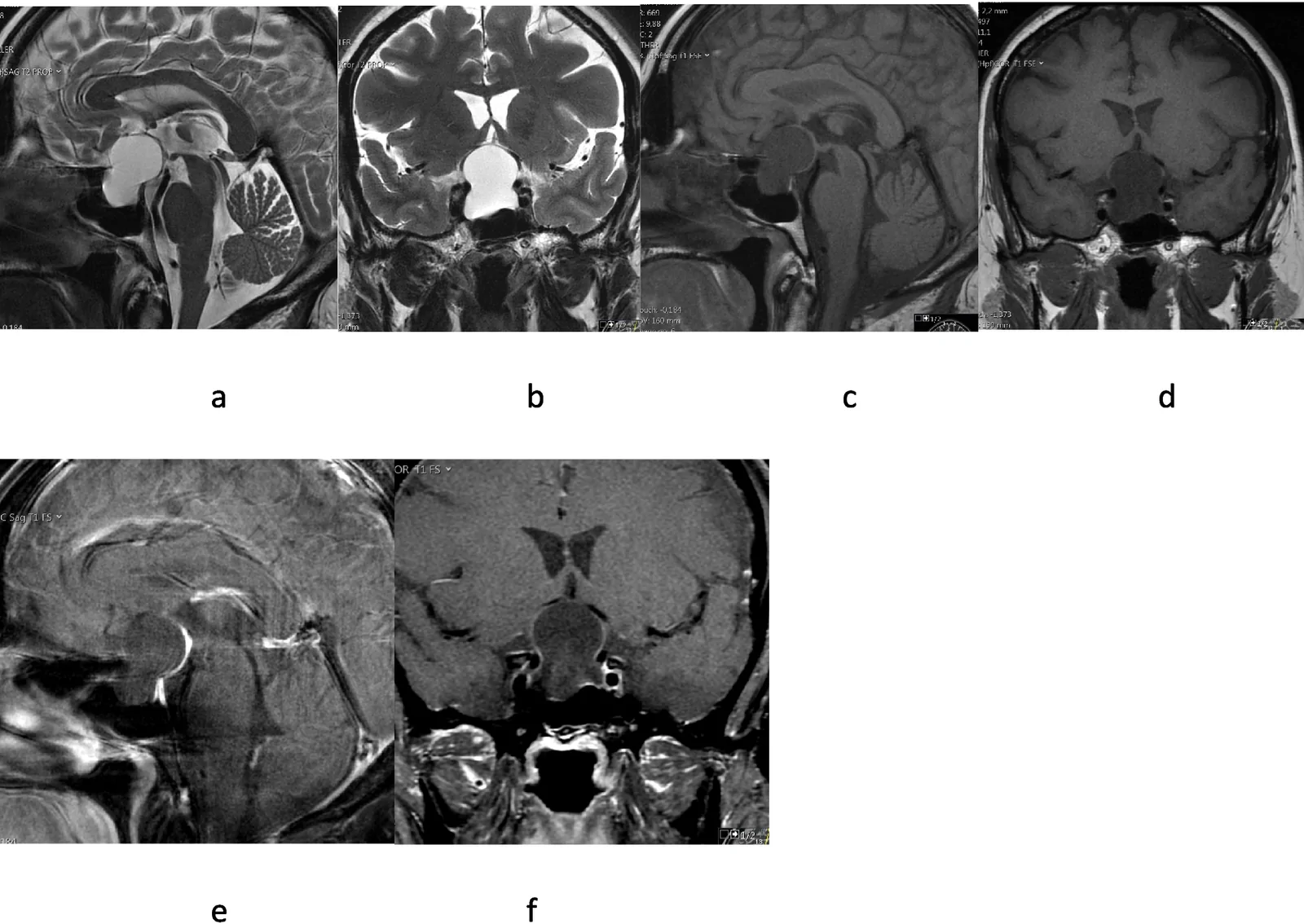
The case of a 46-year-old female with a surgically confirmed arachnoid cyst. Sagittal and coronal planes of T2- and T1-weighted images (**a**-**d**) reveal a large and well-delineated sellar lesion with a significant suprasellar extension. The signal intensity of the lesion is similar to that of CSF. The lesion has a thin wall with no enhancement on the sagittal and coronal contrast-enhanced T1-weighted images (**e**, **f**). Note that the pituitary gland and stalk appear flattened and stretched over the posterior boundary of the cyst

Distinguishing intrasellar arachnoid cysts from other types of lesions relies on specific MRI characteristics. An intrasellar arachnoid cyst is a purely cystic lesion with MRI signals mirroring those of cerebrospinal fluid (CSF). Notably, there is no contrast enhancement or calcification present. On MRI scans, intrasellar arachnoid cysts exhibit hypointensity on T1-weighted images and hyperintensity on T2-weighted images [189]. Key MRI features that can help diagnose an intrasellar arachnoid cyst are the presence of a cystic intrasellar lesion extending into the suprasellar region. These lesions typically exhibit a balloon-shaped, regular morphology and appear iso- or slightly hyperintense in comparison to CSF (Fig. 17). Importantly, there is no evidence of invasion into the cavernous sinus. The accurate diagnosis of intrasellar arachnoid cysts on preoperative imaging is significant for surgical management. Some authors prefer a transcranial surgical approach due to concerns of CSF leakage associated with the transsphenoidal route.

## Management of RCCs

### Medical treatment

Clinical observation with serial MRIs may be practiced for the patients with asymptomatic and small RCCs. Pituitary dysfunction should be managed by replacement of the deficient hormones in accordance to relevant guidelines [190]. Patients who suffer from galactorrhea and/or hypogonadal symptoms secondary to hyperprolactinemia may be treated with dopamine agonists. There have been a few case reports that drew attention to possible involution of RCCs with glucocorticoid therapy [139, 191, 192]. Furtado et al. proposed a trial of glucocorticoids in patients without visual disturbances and who are not suitable for surgery, provided that their RCCs are hypo-to-isointense RCCs on T1-weighted images [139]. There is need for further evidence on the effects of glucocorticoids over the long term clinical course of RCCs [192].

### Surgical treatment

An RCC might not change during the course of a patient’s life. For silent or smaller cysts with moderate symptoms, conservative therapy and MRI follow-up are appropriate, especially in older or younger individuals without fertility. For larger cysts with compression-related symptoms, surgery is recommended. However, surgery is done mainly when the lesion cannot be distinguished from a pituitary adenoma. Most of the time, RCCs are asymptomatic, but they have been known to cause pituitary dysfunction and eye problems, such as bitemporal hemianopsia, which is a defect in the visual field [193]. Most symptomatic RCCs should have the cyst wall aspirated and biopsied [194]. The initial option is transsphenoidal surgery because of its reduced risks of morbidity and mortality; however, the choice of method relies on where the RCC and remnant pituitary gland are.

#### Transsphenoidal approach

For lesions that are located below the sellar diaphragm, the transsphenoidal avenue is highly practical and successful. As the diaphragm is still intact, even if the tumor spreads over the level of the sella, it won’t adhere to the optic chiasm or hypothalamus. The transsphenoidal approach may thus be used to successfully remove these lesions [195]. The endonasal transsphenoidal approach is excellent for reaching lesions restricted to the paramedian sellar and suprasellar areas medial to the carotid arteries and inferior to the subchiasmatic space. The transsphenoidal technique provides better surgical field visibility, minimizes the need for brain retraction, and is the least traumatic nasal approach to the sella turcica.

In surgical management of intrasellar cysts, only partial removal of the cyst wall through a simple transsphenoidal route and cyst drainage is recommended. This approach reduces the risk of pituitary, hypothalamic, or visual complications and aseptic meningitis [196–198]. Although a tiny hole in the bone and dura might theoretically be used to drain a cyst, a wider dural aperture is preferred as it facilitates continual cyst drainage and may even keep the RCC from recurring.

The best surgical approach for treating RCCs and protecting the posterior pituitary gland and pituitary stalk involves resecting a small part of the anterior cyst wall for use as a diagnostic specimen, followed by broad fenestration of the capsule [131, 199–201]. While some surgeons prefer the endoscopic transsphenoidal approach, others prefer the microsurgical transsphenoidal approach. The microsurgical transsphenoidal method is common and efficient because of its midline approach, extensive exposure of the anterior wall of the sella, potential for lateral extension and exposure of the cavernous sinus, and the unrestricted manipulation of instruments. The microscope allows the surgeon to use both hands freely with sufficient illumination in the depth. Generally, the right nostril is used for the transsphenoidal approach. The procedure is carried out beginning with a delicate dissection of the septal mucosa around the anterior quadrangular cartilage or the tip of the vomer bone under local anesthetic (xylocaine with epinephrine). The submucosal incision is extended beyond the septal cartilage and up to the level of the vomer with a Penfield dissector. Even minor holes near the intersection of the cartilaginous and bony septa should be avoided in order to preserve the integrity of the mucosa. Access to both sides of the vomer and the perpendicular plate of the ethmoid is possible as the quadrangular cartilage is moved to the opposite side. The remaining midline bony structures are removed with pituitary rongeurs. The mucosa is retracted laterally, and the sphenoid crest is midline in a bivalve nasal speculum. Superior dissection is continued to liberate the mucosa from the quadrangular cartilage to the vomer inferiorly and the perpendicular plate of the ethmoid posteriorly. These movements reveal the rostrum of the sphenoid sinus. To reach the sinus mucosa and floor of the sella turcica, the anterior aspect of the sphenoid sinus wall may be removed with a chisel and Kerrison rongeurs.

Neuronavigation is used to confirm the precise placement of the midline, sella and the limitations of the exposure. The midline septi separating the sinus into two or more cavities are located and eliminated with pituitary rongeurs. An air drill can be used for a sinus that is not sufficiently pneumatized. The sinus mucosae are fully exenterated, and the front wall of the sella is exposed in great detail during surgery. The anterior sellar wall is then extensively excised, first with a chisel and afterwards with rongeurs.

Some surgeons prefer the endoscopic transsphenoidal approach for surgical removal of the RCC. The two steps of the usual surgical method are nasosphenoidal and sellar. The goal of the nasosphenoidal phase is to maintain the sinonasal architecture and function while establishing a sufficient surgical channel in the posterior nasal cavity. The head of the middle turbinate is displaced laterally to open the space between it and the nasal septum after the endoscope has been inserted into the nasal cavity. The endoscope reaches the choana as it moves posteriorly. A broad sphenoidotomy is carried out after the sphenoid ostium is located. This structure is often situated along the spheno-ethmoidal recess, about 1.5 cm above the roof of the choana and posterior to the superior turbinate. To prevent artery injury and subsequent bleeding, caution must be used in the inferolateral direction, where the sphenopalatine artery and its primary branches are located. The inter- and intrasinusal septa are then removed, exposing the sellar floor, clivus, carotid prominences, opticocarotid recesses, and planum sphenoidale. A transtuberculum extended technique might be the best choice to locate the cyst and maintain normal gland function in patients with an RCC restricted to the suprasellar area. Less commonly, large retrosellar and retroclival RCCs may need a transclival approach. Although a tiny hole in the bone and dura might theoretically be used to drain a cyst, a wider dural aperture is preferred as it is facilitates cyst drainage and may even prevent RCC regrowth.

Using the microscopic or endoscopic transsphenoidal approach, an anterior sellar wall incision is followed by a cruciate incision in the dura. When the cyst capsule is opened, the mucinous interior often protrudes under the low pressure. Subsequent drainage may be helped by using a tiny suction. Despite the rare high vascularity in this area, bleeding can be controlled with hemostats such as Gelfoam and Surgicel. For smaller cysts, it may be simpler to obtain a tiny portion of the front cyst wall for pathological investigation before rather than after drainage. A tiny round, angled curette may help to deliver the contents of RCCs with more fibrous, proteinaceous, or waxy components.

The posterior pituitary gland is directly posterior to the cyst wall in typical RCCs in the pars inter-media, and the surgeon must take special care to prevent damage to this part of the gland. Full excision of the cyst wall is not usually advised as it has been linked to a greater incidence of postoperative diabetes insipidus.

The surgeon should check for signs of an intraoperative CSF leak after the cyst has been completely drained. This check may be aided by doing a Valsalva maneuver. Alcohol cauterization should never be utilized if there is an intraoperative CSF leak as it has not been shown to lower RCC recurrence rates. If there is no CSF leak, rebuilding the sellar floor is not advised as this would prevent the cyst from continuously draining. If an intraoperative CSF leakage is found, it may be repaired with either an autologous belly fat graft and sellar floor buttress or a dural substitute and fibrin glue. The vast majority of these lesions do not need a pedicled nasal flap or lumbar drain. Similarly, regular nasal packing is not done [131, 199–201].

Although the transsphenoidal approach is believed to be the best for RCCs, there are still several potential disadvantages. First, the residual normal gland is situated in the path of the transsphenoidal route. Usually, this avenue necessitates an incision that may increase the risk of postoperative hypopituitarism due to additional trauma to the already compromised gland. This is true even for some intrasellar RCCs with a suprasellar extension. Similarly, a transtuberculum or transplanum approach may be employed to reach suprasellar lesions without passing through the pituitary gland; this technique involves opening the suprasellar cistern, which requires surgical repair. Finally, some investigators have hypothesized that packing the cyst chamber too tightly to stop a CSF leak increases the likelihood of recurrence.

Endoscopic aid, which may increase visibility in the surgical field, further strengthens this advantage. This method's primary flaw is the lack of room for manipulation because of the tiny bone window [202].

Challenges for transsphenoidal surgery include lesion extension and specific traits such as a significant suprasellar extension with a small diaphragma sellae hiatus, lateral and retrosellar extensions, brain invasion with edema, vasospasm of the circle of Willis arteries, and encasement or invasion of the optic pathways and optic foramina. A relative contraindication to the transsphenoidal technique is sinusitis as it may cause infection to move to the brain.

Complete cyst decompression can be achieved in up to 97% of patients, and among those who arrive with visual impairment, vision improves in 83% to 97%. Overall, 71% of patients report an improvement in their headache, while 33% to 94% show an improvement in endocrinopathy [193]. Cysts recur with a frequency of 19% to 28% [129]. Although data is limited, there may be an association between the radiological location and recurrence rate, which was significantly higher in RCCs located in a lateral position than those located in midline position [203]. With a larger risk of endocrine disruption, an aggressive radical resection may result in a low recurrence rate [204, 205].

Surgery-related problems include CSF rhinorrhea (7% of patients), diabetes insipidus (4%), and meningitis (4%) [7, 16, 17]. A perforated nasal septum is a rare surgical consequence. Even in the absence of a fever, this complication should be considered when radiologic traits suggest that an abscess may develop in a cyst. Surgery and antibiotics are effective treatments for the majority of patients. Cysts contents may leak into the subarachnoid space, which may cause aseptic meningitis. Complete excision of the cyst wall is the recommended line of treatment in cases of recurrence. When this therapy is administered, recurrence is rare [5, 129, 194].

#### Transcranial approach

A transcranial approach is recommended for completely suprasellar cysts [206]. When the cyst is only suprasellar or the remaining pituitary gland is anteriorly and inferiorly displaced, a craniotomy is used to prevent further impairment of residual pituitary gland function [207]. The absence of a pituitary gland incision for solely or mainly suprasellar RCCs is a vital advantage of the transcranial technique over the transsphenoidal route. Depending on the location of the cyst, the pterional, supraorbital keyhole, subfrontal, or transventricular can be applied.

### Radiotherapy

A potential new therapeutic option for RCCs is radiotherapy, which may have small risks in selected cases. Endocavitary irradiation and stereotactic radiosurgery are the two kinds of radiotherapy used in the treatment of RCC. Cytostatic agents or radioactive solutions can be injected into the cyst, but the literature has only one example of this treatment for an RCC. Moringlane, et al., applied intracavitary rhenium-186 to an RCC in 2001. In this case, symptomatic recurrence of a histologically confirmed intra- and suprasellar RCC was observed 4 months after transsphenoidal microsurgery. The patient underwent endocavitary irradiation with colloidal rhenium-186 through a catheter implanted before treatment. The calculated dose of 4.4 Gy was able to stop the production of cyst fluid. The patient was followed for more than 13 months afterward. Initially, the cyst was 3 × 3 × 4 cm but was reduced to 1.1 × 1.06 × 1.2 cm after treatment, and fluid production decreased from 25 to 30 ml to zero in the 2 months after treatment [208]. RCCs recur after surgical treatment at a frequency of 10% to 30% [209–212]. With the risk of recurrence, the volume of the cyst appears to relate to some features of the lesion, such as rapid growth, a suprasellar location, and the presence of squamous metaplasia in the cyst wall. When a cyst recurs, the patient requires further surgical treatment, but with adhesions from the previous surgery and the difficulty in eradicating the lesion, further surgical intervention creates new risks [119, 213–216]. RCCs are histologically similar to and embryologically related to craniopharyngiomas [217]. Studies on the efficacy of stereotactic radiosurgery in the treatment of patients with recurrent craniopharyngioma after primary surgical resection have been reported [218]. Stereotactic radiosurgery poses less risk to optic tracts and the hypothalamic-pituitary axis than open surgical treatment. In particular, the risk of radiation-induced optic neuritis is less than 1%, and the single-fraction spot dose is less than 10 Gy [219]. Very few studies report the use of sterotactic radiosurgery (SRS) for patients with an RCC. Yu, et al., use the Gamma Knife (mean 13.4 Gy) in seven patients with a symptomatic RCC diagnosed through typical MR imaging. They were followed for 38.6 months on average. All symptoms resolved within 3 to 6 months after SRS and endocrine dysfunction did not develop in the follow-up period. MR imaging showed that the RCCs disappeared completely in five patients and significantly shrank in the other two. No recurrence was observed during the follow-up period [220]. In another study by West, et al., radiosurgery was used in five female patients who had undergone at least two previous operations. The median dose of SRS was 12.5 Gy as the prescribed dose to the 50% isodose line. The overall mean follow-up was reported as 34.2 months. During this period, three patients had complete remission and one experienced regression. There was no shrinkage in one patient but the lesion was considered stable. No neurological, endocrinological, or visual complications attributable to SRS were observed during the follow-up period [221]. In another study by Manzoni, et al., the Gamma Knife was used in a patient with an RCC because of early regrowth of the cyst despite two surgeries. Radiosurgery was applied with a prescription dose of 12 Gy to the 50% isodose line. Three years later, the patient was asymptomatic and showed no abnormalities of the hypothalamic-pituitary axis or other visual changes. Stereotactic radiosurgery caused the cyst to decrease in volume [222]. Kondziolka, et al., published their results from 25 RCC patients who underwent multicenter SRS from 2001 through 2020. Diagnosis was based on imaging or histopathology. Four patients underwent SRS based on radiological diagnosis without surgery. The other 21 were treated with salvage SRS. The median clinical follow-up was 6.5 years and the mean dose administered was 12 Gy. Overall control was achieved in 19 patients (76%), and 4 relapses required further intervention. The mean time to relapse was 35.6 months. Vision improved in 14 of 15 patients (93.3%) patients, and no new visual deficits occurred after SRS. All 3 patients with pre-treatment hyperprolactinemia recovered after SRS. A new endocrinopathy from SRS was recorded in 5 of the 25 patients (20%). One patient with visual impairment before treatment recovered [223]. Agarwalla, et al., described the cases of 6 patients, 83.3% of whom were female, who underwent SRS for recurrent RCCs from 1994 to 2015. All patients had initial postoperative recovery but subsequently developed multiple symptomatic recurrences. The median number of surgical drainage procedures before radiotherapy was 3. A total of 3 patients received LINAC-based SRS and 3 patients received proton-based SRS. Treatment doses were 45 Gy over 25 fractions (n = 5) and 50.4 Gy over 28 fractions (n = 1). The median follow-up period was 69 months (range 24–154 months). During the follow-up period, RCC stabilization was achieved in 2 patients, although additional drainage procedures were required. New hypothyroidism and hypoadrenalism developed in only 1 patient [224]. In conclusion, the use of SRS in patients with RCC recurrence seems an effective treatment with small risks compared to open surgery.

## Future directions

Despite dramatic increase in pathologic and radiologic facilities, differential diagnosis of RCCs might still be challenging in some cases. It might not be straightforward to differentiate a RCC from a cystic adenoma or craniopharyngioma radiologically. Recent advances in radiomics and deep learning emerged as a potential method to increase diagnostic accuracy of sellar masses [225]. RCCs were successfully differentiated from cystic craniopharyngiomas by use of a machine learning model in a recent study [226]. Moreover, the method of combining pathologic and genetic data with radiomics to predict histologic subtypes of pituitary lesions had promising results [227, 228]. Future studies designed with more precise machine learning models might be expected to be game-changers in the area of diagnosis of cystic sellar masses. On the other hand, molecular studies provided new insights regarding the pathogenesis of RCCs, and new therapeutic modalities with targeted molecular agents might be a promising option in difficult RCCs.

## Data Availability

No datasets were generated or analysed during the current study.
